# Protocols for the Evaluation of Neurodevelopmental Alterations in Rabbit Models *In Vitro* and *In Vivo*

**DOI:** 10.3389/ftox.2022.918520

**Published:** 2022-07-22

**Authors:** Laura Pla, Britta Anna Kühne, Laia Guardia-Escote, Paula Vázquez-Aristizabal, Carla Loreiro, Burkhard Flick, Eduard Gratacós, Marta Barenys, Miriam Illa

**Affiliations:** ^1^ BCNatal-Barcelona Center for Maternal-Fetal and Neonatal Medicine (Hospital Clínic and Hospital Sant Joan de Déu), Fetal i+D Fetal Medicine Research Center, IDIBAPS, University of Barcelona, Center for Biomedical Research on Rare Diseases (CIBER-ER), Barcelona, Spain; ^2^ GRET, INSA-UB and Toxicology Unit, Pharmacology, Toxicology and Therapeutical Chemistry Department, Faculty of Pharmacy, University of Barcelona, Barcelona, Spain; ^3^ Department of Psychology, Faculty of Psychology, Universitat Rovira i Virgili, Tarragona, Spain; ^4^ Department of Toxicology, NUVISAN ICB GmbH, Berlin, Germany

**Keywords:** neurospheres, object recognition test, behavioral ontogeny, Skinner box, Golgi staining, open field, perineuronal nets, neurodevelopment

## Abstract

The rabbit model is gaining importance in the field of neurodevelopmental evaluation due to its higher similarity to humans in terms of brain development and maturation than rodents. In this publication, we detailed 14 protocols covering toxicological relevant endpoints for the assessment of neurodevelopmental adverse effects in the rabbit species. These protocols include both *in vitro* and *in vivo* techniques, which also cover different evaluation time-points, the neonatal period, and long-term examinations at postnatal days (PNDs) 50–70. Specifically, the protocols (P) included are as follows: neurosphere preparation (GD30/PND0; P2) and neurosphere assay (P3), behavioral ontogeny (PND1; P4), brain obtaining and brain weight measurement at two different ages: PND1 (P5) and PND70 (P12), neurohistopathological evaluations after immersion fixation for neurons, astrocytes, oligodendrocytes and microglia (PND1; P6-9) or perfusion fixation (PND70; P12), motor activity (P11, open field), memory and sensory function (P11, object recognition test), learning (P10, Skinner box), and histological evaluation of plasticity (P13 and P14) through dendritic spines and perineuronal nets. The expected control values and their variabilities are presented together with the information on how to troubleshoot the most common issues related to each protocol. To sum up, this publication offers a comprehensive compilation of reliable protocols adapted to the rabbit model for neurodevelopmental assessment in toxicology.

## Introduction

The rabbit model has become increasingly popular in neurodevelopmental studies within the translational medicine research field because it is a perinatal “brain developer”, similar to humans (Derrick et al., 2004; Eixarch et al., 2012; Workman et al., 2013). In the toxicology field, the most commonly used species is the rat. However rabbit is the preferred non-rodent species for prenatal developmental toxicity studies as detailed in OECD Test Guideline (TG) 414 (OECD, 2018). Besides that, in the OECD TG 426 (OECD, 2007) for testing the effects of chemicals on developmental neurotoxicity it is stated that developmental neurotoxicity studies can be conducted separately, or in addition to a prenatal developmental toxicity study (OECD TG 414 (OECD, 2018)). The OECD TG 426 indicates that the rat is the preferred test species to perform *in vivo* studies but states that other species can be used when appropriate (OECD, 2007). In that case, the use of other species should be justified based on toxicological, pharmacokinetic, or other data and should include the availability of species-specific postnatal neurobehavioral and neuropathological assessments.

The adaptation of protocols regularly used to evaluate developmental neurotoxicity in rats to rabbits could be useful in specific cases where the rabbit could mimic human conditions better than the rat, especially if it is taken into account that the OECD TG 426 indicates that if there is an earlier test raising concerns on developmental neurotoxicity the species/strain that raised this concern should be considered for the developmental neurotoxicity assay instead of the rat (OECD, 2007). From several review articles comparing the effects of test compounds in the OECD TG 414 performed in rats and rabbits, it has been demonstrated that there is an overlap of detectable effects in these species (Theunissen et al., 2016; Theunissen et al., 2017; Teixidó et al., 2018). However, there is a significant proportion of compounds with embryo-fetal developmental toxicity only detected in one of both species. The proportion of chemicals or drugs whose developmental toxic potential was only described in rabbits and not in rats was around 13% in several large-scale comparative studies (Theunissen et al., 2016; Theunissen et al., 2017; Teixidó et al., 2018). For all these reasons, it is plausible that the rabbit species becomes relevant for the evaluation of developmental neurotoxicity in particular cases. For this purpose, all developmental neurotoxicity protocols would need to be adapted to the new species, such as the moment of postnatal behavioral evaluation, behavioral equipment sizes, the inclusion of species-specific behaviors, scoring systems, antibodies used in histopathological analyses, incubation times, etc., and the reliability and sensitivity of the rabbit species to detect developmental neurotoxicity all would need to be documented.

The preparation of rabbit neurospheres from surplus control rabbit pups from OECD TG 426 (OECD, 2007) for later developmental neurotoxicity testing *in vitro* of other compounds or for mechanistic studies *in vitro* would help in the reduction of animal numbers used for neuropathological evaluations, although behavioral studies would still need to be tested *in vivo*.

The main advantages of using the rabbit species in developmental neurotoxicity studies are 1) a higher similarity to humans than rodent species regarding brain development and white matter maturation-timing, since they undergo perinatal brain development and begin myelination postnatally (Derrick et al., 2004; Eixarch et al., 2012; Workman et al., 2013), 2) a more complex brain structure than rodents with a higher ratio of other cells/neurons (Herculano-Houzel et al., 2011; Ferraris et al., 2018), and 3) a higher similarity to humans than rodents in terms of extraembryonic membranes, placenta development and circulatory changes during gestation (Foote and Carney, 2000; Carter, 2007; Eixarch et al., 2009). According to the inter-species comparison model developed by Clancy, Darlington, and Finlay 2001 and Workman et al., 2013 to predict the “precocial score” for neurodevelopment, rabbit species have a precocial score at birth (0.537) more similar to humans (0.654) than rats (0.445) or mice (0.408). In addition to that and from a practical point of view, several tools are facilitating the use of this model for the evaluation of neurodevelopmental alterations: the availability of a rabbit brain atlas since 2013 (Muñoz-Moreno et al., 2013), and the availability of the *in vivo* reference database (ToxRefDB) including a public dataset on endpoints from guideline prenatal developmental toxicity studies in pregnant rabbits (Knudsen et al., 2009; Knudsen et al., 2013) (http://actor.epa.gov/toxrefdb/faces/Home.jsp).

However, performing developmental neurotoxicity studies in rabbits entails some difficulties compared to the commonly used rat species, since the time and cost of the experiments are increased due to a longer gestation period, a higher amount of test compound needed per animal, and to the much larger room space required, among others. If the study follows OECD TG 426, including 20 rabbit litters per group can be logistically limiting, especially considering that a very careful animal housing of reproducing does in individual cages where the nest area is in a separate section of the mother’s living environment is needed to avoid mismothering and to ensure proper development of the litter.

Taking all that into consideration, in this manuscript, we present a first comprehensive approach for the adaptation of protocols for the evaluation of neurodevelopmental adverse effects during the neonatal period and in the long-term period in the rabbit species including behavioral tests and neuropathological evaluations *in vivo* and *in vitro*. Critical steps in the protocols and limitations of the techniques are presented and discussed together with the expected control values and variabilities, as well as with orientation on data interpretation combining the different tests. The 14 protocols proposed allow the assignment of rabbit fetuses or pups to a combination of tests depending on the interest of the researchers and cover most of the endpoints currently required for developmental neurotoxicity in OECD TG 426 (OECD, 2007). Explanations on the exposure of the does during gestation and evaluation of maternal toxicity are not a matter of this protocol series, since they are already presented in detail in OECD TG 414 for prenatal developmental toxicity studies (OECD, 2018).

## Protocols

All procedures should comply with applicable governmental and institutional regulations for the use of laboratory animals in research.

**FIGURE 1:**
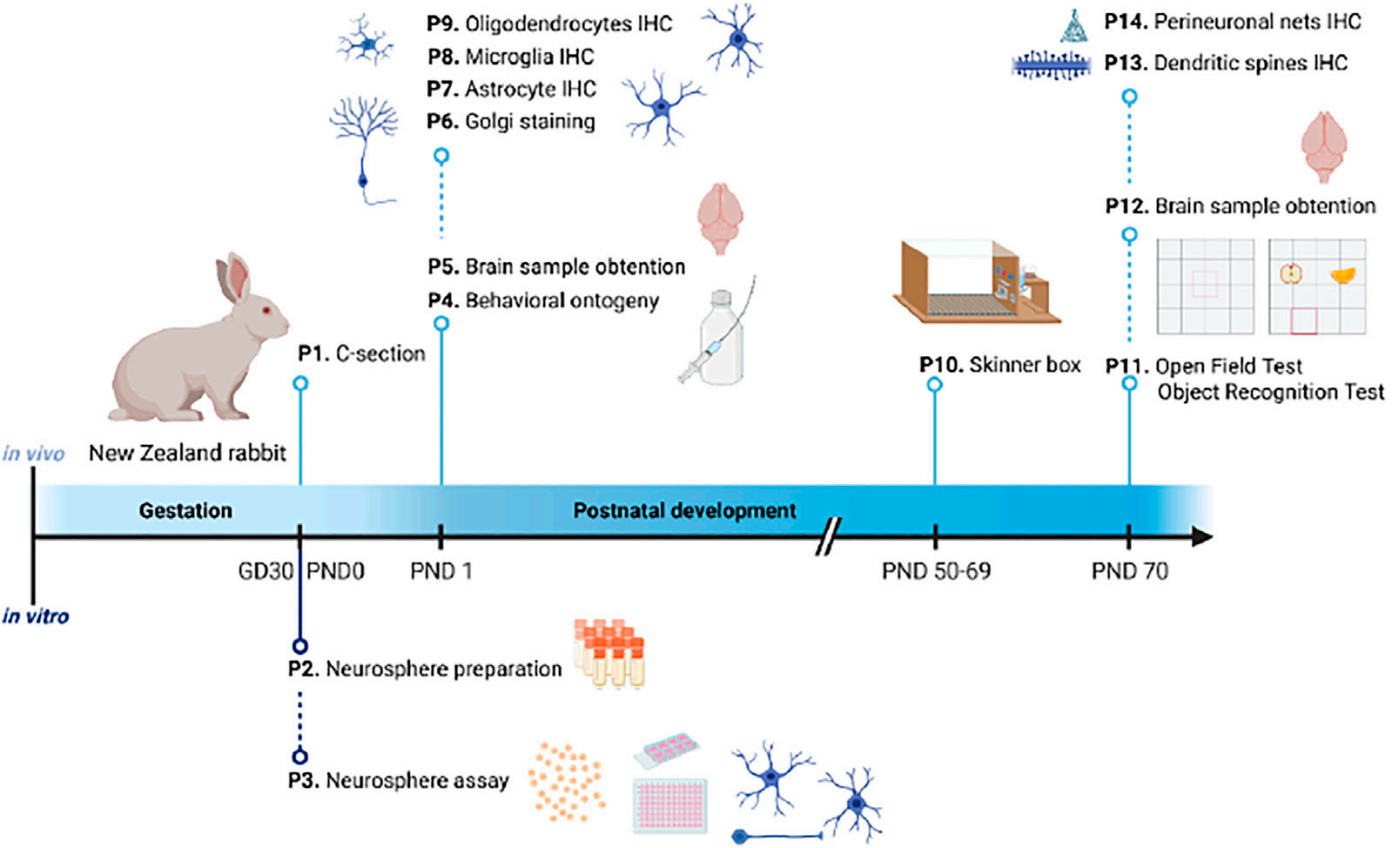
Schematic representation of the timeline organization of the 14 protocols presented in this publication. Under the timeline, protocols for *in vitro* testing; above the timeline, protocols for *in vivo* testing. GD: gestational day; IHC: immunohistochemistry; P: protocol; PND: postnatal day.

An overview of the 14 protocols detailed in this work is presented in Figure 1. On GD30/PND0, New Zealand rabbit fetuses or pups are selected from the different study groups and are assigned for *in vivo* endpoint assessments. The *in vivo* protocols presented cover toxicological relevant endpoints in neurologic development required or optional in OECD TG 426 (OECD, 2007) ranging from basic behavior ontogeny to complex operant conditioning or sensory behavior, as well as neurohistopathological evaluation of different cell types and cell characteristics. Specifically, they include brain weight measurement at two different ages: PND1 (P5) and PND70 (P12), neurohistopathological evaluations after immersion fixation (PND1; P6-9) or perfusion fixation (PND70; P13 and 14), behavioral ontogeny (P4), motor activity (P11, open field), memory and sensory function (P11, object recognition test), learning (P10, Skinner box), and plasticity (P13 and P14). Protocols to evaluate relevant endpoints required in OECD TG 426 (OECD, 2007) which are not included in this first approach are neurohistopathological evaluation of peripheral nervous system (PNS) at PND70, global behavior assessment using a functional observational battery (FOB) or modified Irwin test, sexual maturation evaluation, and assessment of other developmental landmarks (optional in TG 426).

Offspring are randomly selected from within litters for the different neurotoxicity evaluations. Among the protocols included in the present approach, there are different possibilities to assign pups to these neonatal (PND1) and long-term examinations (PND50 to 70), but to avoid interferences among tests, the recommended assignment is as follows:


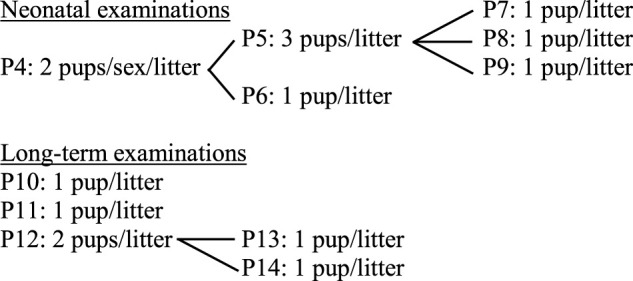


**FIGURE 2:**
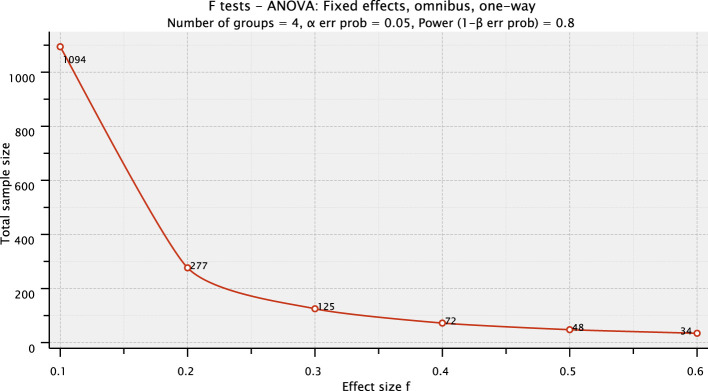
G*POWER plot of the total sample size (y-axis) depending on the effect size f expected (x-axis) after establishing the parameters: number of groups= 4; α error probability= 0.05, and power (1-β error probability) = 0.80. The total sample size indicated includes the four groups. The effect size f range plotted covers small (0.1), medium (0.2), and large (0.4 or higher) effects for ANOVA, as established by Cohen (1969).

The number of litters to be assigned to each group depends on the aim of the study, the statistical power desired, and the effect size expected. Using the statistical power analysis tool G*POWER (Faul et al., 2007; Faul et al., 2009) and considering a typical assay with four groups (1 control and three treatment groups), whose results would be analyzed using a one-way ANOVA test and establishing an α error probability of 0.05 (*p* ≤ 0.05; Type I error [false positive] less than 5%) and a power of 0.80 [1-β error probability; Type II error (false negative) less than 20%], if the expected effect size f is large (0.4), 19 litters per group would be recommended, similarly to the OECD TG 426 recommendation of 20 litters per group; but if a larger effect size f is expected (0.57) the requirements decrease to 9 litters per group. Therefore, pilot/screening studies with less than 10 liters per group would be sufficient to detect very large effects, while higher numbers of litters would be needed if a small effect size f is expected (0.1) as exemplified in Figure 2.

In addition to these considerations, litter size standardization (culling) prior to functional endpoints testing is accepted in OECD TG 426 (OECD, 2007); therefore, control pups not selected for *in vivo* evaluations can be assigned to Protocol 2 (neurosphere preparation) for the isolation of neural progenitor cells (NPC) which can be frozen for future *in vitro* testing in the neurosphere assay (Protocol 3).

### Protocol 1: Cesarean Section

There are two options to obtain the fetuses or pups for protocols from 2 to 14, by natural delivery and proceed with all the protocols at the PND indicated in Figure 1 or to obtain them by cesarean section and proceed in the same way. This protocol describes the basic procedures to perform a cesarean section on GD30 in New Zealand rabbits. The protocol is not strictly necessary for toxicological studies, but it can be necessary in case a different condition needs to be induced during gestation in the two different uterus horns, such as intra-uterine growth restriction induced in one of the uterus horns by ligation of uteroplacental vessels, and to allow the identification of the case and control pups. As the control values presented in the results section are from pups obtained by cesarean section, this protocol is also included in this collection. If the cesarean section step can be avoided, the number of animals needed will be reduced since after cesarean section pups need to be assigned to a nursing mother for further development.

#### P1 Materials and Equipment

- Data collection sheets.
- Monitor for blood pressure, heart rate, and blood oxygen level.
- Gauzes.
- Scalpel blade number 24 and scalpel handle.
- Atraumatic forceps.
- Dissection scissors.
- 1 syringe (2.5 ml).
- Surgical table: bandage to hold the animal, soaker, surgical drape, and iodine.
- Venous catheter.
- Fixing paper for the catheter.
- Three-way catheter.
- Incubator.
- High-precision flow regulator.
- Medication for anesthetic induction based on ketamine and xylazine: (see Supplementary File S1: Reagents and Solutions list).
- Medication for anesthetic maintenance based on ketamine and xylazine: (see Supplementary File S1: Reagents and Solutions list).

#### P1 Methods

##### P1.1. Before the Surgery

1| Prepare all the medication before the cesarean section.
2| Anesthesia induction:
  a) Administer the anesthetic medication i.m.
  b) Bring the rabbit to the surgical table, fix the limbs and set the monitor for blood pressure, heart rate, and blood oxygen level.
  c) Place the catheter in the lateral vein of the ear, fix it, and place a three-way catheter. Administer a small volume of propofol to check the permeability of the catheter.
  d) Anesthesia maintenance:
  i. Inhalation: place a mask in the airway, connected to the machine, and then start the administration of 2 L of O_2_ and 1.5–2 ml of isoflurane.
ii. Intravenous: add 4 ml ketamine and 3 ml xylazine in 100 ml saline solution and connect the high-precision flow regulator adjusted to administer 30–40 ml/h (corresponds to the necessary dose for a 4–5 kg animal).

##### P1.2. During the Surgery

1| Cover the skin with iodine, place a sterile surgical drape, and dissect planes until opening the abdominal cavity and identify both uterine corns. All the procedures must be carried out under aseptic conditions.
2| Fetus extraction:
  a) Open the uterine cavity with dissection scissors, extract the fetuses (without ligation of the umbilical cord), and extract the placenta. Once all the fetuses have been removed, administer i. v. the first dose of pentobarbital 40 mg/kg, and afterward the second dose of 360 mg/kg for euthanization.
  b) Identify each pup with holes in the ears.
  c) Start cardiopulmonary resuscitation maneuvers in the incubator area and maintain body heat.

##### P1.3. After the Surgery

1| Remove pups from the incubator (maintaining body heat); weigh each newborn and annotate the information. Put them with a nursing mother, all of them at the same time.

### Protocol 2: Rabbit Neurosphere Preparation

On GD30/PND0 control fetuses/pups which are not needed in any *in vivo* evaluation group can directly be assigned to the preparation of neurospheres. The aim of this protocol was to generate a cell suspension from rabbit GD30/PND0 brains and to freeze it in cryovials at −80°C until its use in protocol 3. The cell suspension obtained in protocol 2 will be the starting material for the generation of neurospheres.

#### P2 Materials and Equipment

- Heater and water bath (37°C).
- Binocular microscope.
- 15 and 50 ml centrifuge tubes.
- Petri dishes (60 and 90 mm ± poly-HEMA).
- Pipettes (1000 μl, 100 μl, and 10 μl).
- Preparation cutlery: forceps and scissors.
- Centrifuge for 15 and 50 ml tubes.
- Syringes (20 ml).
- Syringe filters (sterile membranes with 0.22 µm pore size).
- Cell strainer (sterile, 100 µm).
- Cryo-freezing container filled with isopropyl alcohol.
- Cryogenic storage vials.
- DMSO.
- Sterile PBS.
- MEM.
- DMEM.
- B27 proliferation medium.
- Tissue digestion solution (see Supplementary File S1: Reagents and Solutions list).
- Ovomucoid solution (see Supplementary File S1: Reagents and Solutions list).
- Papain.
- DNaseI (dilute 4 mg/ml in MEM).
- Trypsin inhibitor (dilute 1 mg/ml in DMEM).
- BSA (10% in PBS).
- Fetal bovine serum.
- Class II biological safety cabinet.
- Vial with Rabbit NPC (not older than 6 months).
- Human recombinant fibroblast growth factor (rhFGF).
- B27 proliferation medium (Supplement B27 (50x) serum-free).
- Freezing medium (see Supplementary File S1: Reagents and Solutions list).
- ROCK inhibitor Y-276322.
- Tissue chopper.
- Razor blade (5 cm) stored in 70% ethanol.

#### P2 Methods

##### P2.1. NPC Isolation

1| Fill one tissue culture dish per two brains with sterile 1x PBS (prewarmed) and one dish per two brains with MEM (prewarmed).
2| Fill one 15 ml tube with 1 ml MEM for each half brain.
3|Transfer the pup’s head into a PBS-filled tissue culture dish.
4| Remove skin and cartilage with forceps to uncover the brain. Transfer the brain from the skull base into a tissue culture dish filled with MEM.
5| Remove meninges and olfactory bulbs from the brain, cut the brain (sagittal) in two halves, and place each one in one of the tubes with 1 ml MEM (prepared in step 2).
6| Repeat steps three to five for all brains.
7| Prepare tissue digestion solution, for each half brain (calculate for two additional half brains).
8| Cut the brains into small pieces and add 1 ml tissue digestion solution to the tissue, shake the tube gently, and incubate at 37°C, 20 min.
9| Fill one 15 ml tube with 9 ml DMEM (prewarmed) for each half brain.
10| Prepare ovomucoid solution, for each half brain (calculate for two additional half brains).
11| Triturate the tissue gently with a 1000 μl tip (until all cells are dispersed).
12| Add 1 ml ovomucoid solution to each brain-tube to stop the digestion and mix by pipetting.
13| Transfer each single-cell suspension of one brain into one tube with 9 ml DMEM (prepared in step 9).
14| Centrifuge for 10 min at 163 rcf.
15| Discard the supernatant by decanting and resuspending pellet in 1 ml B27 proliferation media (prewarmed).
16| Pool the two half brains of each brain while pouring or pipetting the cell suspension over a cell strainer (100 µm) fitted on a 50 ml tube. Rinse the cell strainer with another 1 ml of B27 media.
17| Centrifuge again the cell suspension for 10 min at 163 rcf.
18| Prepare the freezing medium.
19| Discard the supernatant and gently resuspend the pellet in 1 ml freezing medium.
20| Transfer cell solution to labeled cryovials and directly place it in a cryodevice into −80°C for 24 h.
21| Remove vials from the cryodevice and store the vials not longer than 6 months at −80°C.

##### P2.2. Thawing Rabbit NPC and Forming Neurospheres

Preparation for each vial to be thawed:

•Prewarm 2 × 50 ml of B27 medium in a culture flask for 2 h at 37°C.
•Prepare preconditioned B27 medium + 20 ng/ml rhFGF_2_ + 10 μM ROCK inhibitor Y-276322 (1:1000 from stock solution).
•Fill six 6 cm poly-HEMA coated Petri dishes (6 ml) and one 15 ml centrifuge tube with prepared B27 medium (10 ml).
1| Thaw vials in warm water until the ice starts to melt.
2| Quickly transfer the complete content of the vial to a 15 ml centrifuge tube previously filled with 10 ml of preconditioned B27.
3| Centrifuge at 163 rcf for 10 min.
4| Discard the supernatant.
5|Resuspend spheres gently in 0.5 ml of preconditioned B27 medium and distribute it homogeneously (approx. 10 μl) to the previously prepared 6 cm poly-HEMA Petri dishes (with 6 ml B27).
6| Incubate rabbit NPCs at 37°C and 5% CO_2_.
7| Change half of the medium with fresh B27 medium without the ROCK inhibitor every 2–3 days.
8| After approx. 11 days, spheres should be ready to be chopped for starting experiments.

##### P2.3. Chopping Neurospheres

1| Chop rabbit NPCs 2–3 days to 0.2 mm always before starting an experiment.
2| Prewarm B27 proliferation medium (37°C); add 20 ng/ml rhFGF.
3| Label required Petri dishes (90 mm) previously coated with Poly-HEMA: animal code, P0: thawing date, P1: new passage number, date.
4| Pipette 20 ml of fresh prewarmed B27 into each Petri dish previously coated with Poly-HEMA.
5| Disinfect the tissue chopper with 70% ethanol. Make sure that no ethanol remains on the blade before placing the neurospheres under the chopping arm.
6| Pinch a coverlid of a small Petri dish (60 mm) on the intended position under the chopping arm.
7| Take the razor blade out of a centrifuge tube filled with ethanol. Use one side for a maximum of two times.
8| Screw the razor blade on the chopping arm, align its position, and fixate the blade. Run the chopper to test if the blade is in the right position; otherwise, adjust it again.
9| Set the chopper to 0.2 mm.
10| Gently swirl the Petri dish to bring all neurospheres in the middle, collect and place them on the lid (center) of a small Petri dish (60 mm) with as less medium as possible (If you want to chop neurospheres from the same animal from two or multiple dishes, transfer and pool them first in one Petri dish).
11| Remove the medium surrounding the spheres under the binocular microscope until the pink shimmer can no longer be recognized. This is to ensure that the spheres will be really cut and not just pushed aside by the blade in the liquid film.
12| Pinch the coverlid with spheres below the chopper arm and start (the arm of the blade must be positioned to the right of the neurospheres). After the first run, turn the lid of the Petri dish to 90° and chop again.
13| Place on top of the freshly chopped spheres ca. 500 μl B27 medium with rhFGF and separate them by repeated up and down pipetting using a 1000 μl pipette (avoid producing air bubbles).
14| Subsequently, distribute evenly the cell suspension on the prepared Petri dishes.
15| In case you want to chop several neurosphere cultures from different animals, clean the razor blade with ethanol before each subsequent chopping session.
16| Finally, clean the chopper with 70% ethanol and remove it from the bench.

### Protocol 3: Neurosphere Assay

**FIGURE 3:**
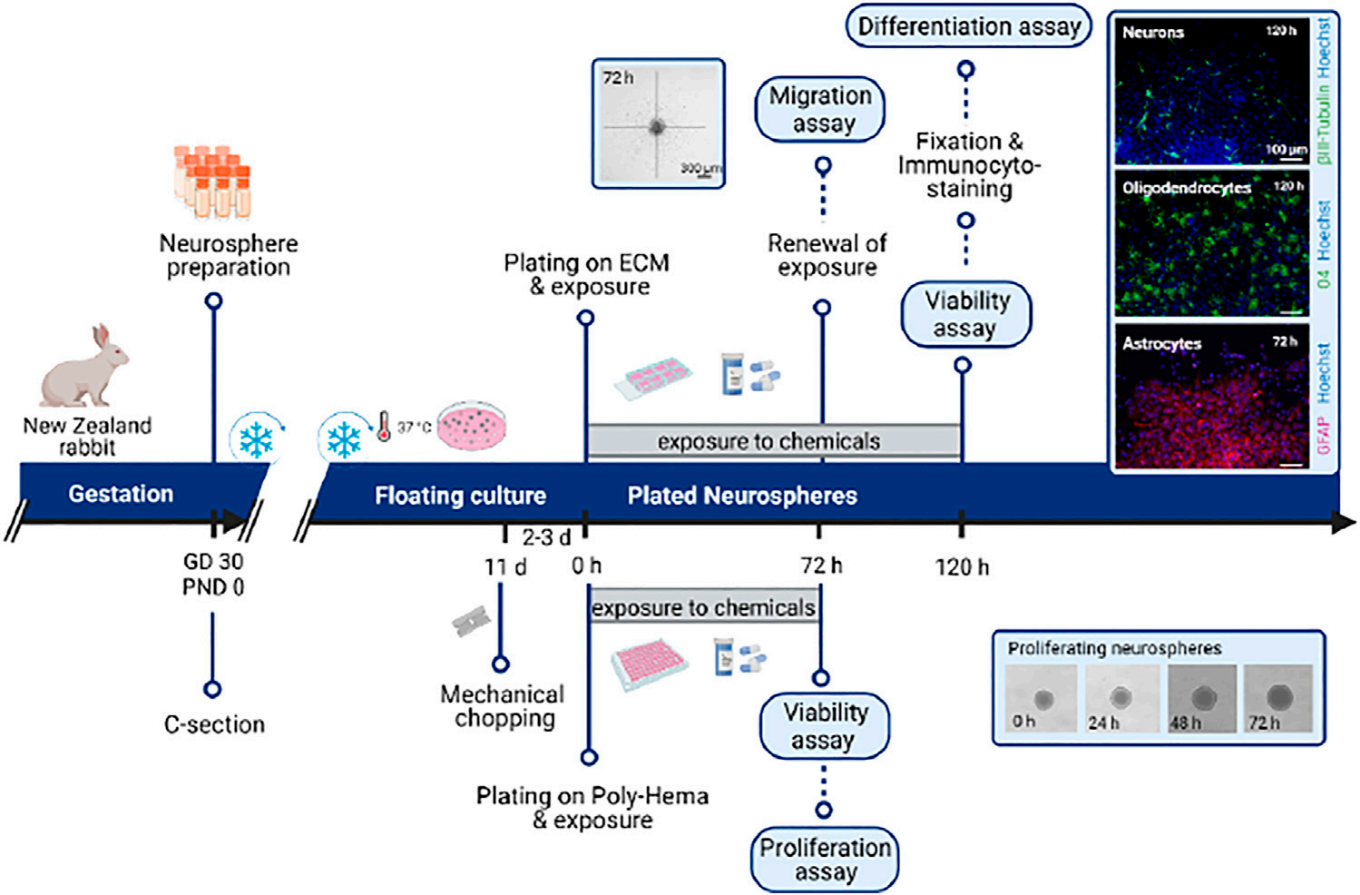
Schematic representation of the timeline corresponding to the procedures detailed in Protocols 2 (rabbit neurosphere preparation) and 3 (neurosphere Assay). ECM: extracellular matrix; GD: gestational day; PND: postnatal day.

This protocol includes all steps needed to perform the whole neurosphere assay with rabbit neurospheres. For a graphical summary of the timeline of the neurosphere assay, see Figure 3. The neurosphere assay allows the *in vitro* evaluation of developmental neurotoxic effects in several endpoints in parallel: differentiation, migration, viability, and proliferation.

#### P3 Materials and Equipment

- Class II biological safety cabinet.
- Cell culture incubator (37°C; 5% CO_2_).
- Laboratory heating oven (37°C).
- Binocular microscope.
- Poly-D-Lysin (PDL).
- PDL solution.
- Laminin.
- H_2_O, deionized and sterile.
- Sterile PBS.
- N2 media.
- 12% paraformaldehyde solution in PBS, pH 7.4.
- Eight-chambered cell culture slides.
- Unsterile PBS.
- PBST 0.5%.
- Mounting medium.
- Goat serum.
- Bovine serum albumin.
- Nucleic acid stain.
- O4 mouse monoclonal antibody IgM (R&D Systems #MAB1326).
- Anti-β-Tubulin III rabbit antibody IgG (Sigma #T2200).
- Anti-Glial Fibrillary Acidic Protein (GFAP) rabbit antibody IgG (Sigma #G9269).
- Goat anti-Mouse IgM Secondary Antibody.
- Goat anti-Rabbit IgG Secondary Antibody.
- Fluorescence plate reader.
- CellTiter-Blue cell viability assay kit.
- CellTiter-Blue reagent (protect from light).
- DMSO.
- B27 w/o (without growth factors) media.
- 96-well plate (clear, U-bottom).
- Poly-HEMA solution (see Poly-HEMA coating in Supplementary File S1: Reagents and Solutions list).
- Solutions to be used as positive controls in the neurosphere assay.

#### P3 Methods

##### P3.1. Neurosphere Differentiation Assay

1| Preparation for cell plating:
  a) Chop neurospheres to a size of 0.2 mm 2–3 days prior to the experiment. Only rabbit neurospheres in passage 1 are used for the differentiation assay.
  b) Prewarm N2 media at 37°C.
  c) Prepare previously coated 8-chamber slides by removing PBS (for PDL-Laminin coating protocol, see Supplementary File S1: Reagents and Solutions list).
  d) Prepare all treatments and control solutions and add 500 μl to each chamber of an 8-chamber slide.
  e) Equilibrate the slide at 37°C and 5% CO_2_ for 20 min.
2| Plating and cultivation of spheres:
  a)Sort the desired amount of 0.3 mm sized spheres from a Petri dish into a new Petri dish (60 mm) with 5 ml of N2 media (37°C).
  b)From the presorted spheres transfer 5 spheres in 10 μl media into one PDL/Laminin coated chamber of an 8-chamber slide. Place spheres in each well (except in the well of the background control) in the same position as the dots of dice in face 5.
  c)Culture the spheres for 3 or 5 days depending on the endpoint at 37°C and 5% CO_2_:
      i. 3 days: GFAP and β-Tubulin III staining
    ii. 5 days: O4 and β-Tubulin III staining
  d)After 3 days in culture, take brightfield pictures from the migration area from each sphere using the microscope and camera to analyze the migration distance (see 3.2. Migration of neurospheres).
  e)For experiments ending on day 3 the viability assay “Cell Titer Blue (CTB) Assay” can be performed.
  f)For experiments ending on day 5, after 3 days feed the spheres by removing half of the media (250 μl) and carefully adding freshly prepared control/treatment solution (prewarmed to 37°C). Continue culturing the slide at 37°C and 5% CO_2_ until day 5 and perform the viability assay “Cell Titer Blue (CTB) Assay”.
  g)Add PFA (12%) in a 1:3 proportion to each chamber (final concentration 4%) and incubate 30 min at 37°C.
  h)Carefully remove PFA.
  i)Wash 3 × 3 min by addition and removal of 500 μl PBS.
  j)Slides can be stored filled with 500 μl PBS/chamber and sealed with Parafilm at 4°C for a maximum of 4 weeks until immunocytochemical staining is performed.
3|Immunocytostaining of neurospheres:
  a)Fixation: see steps g) to j) of step 2 of this protocol.
  b)Washing:
      i. Carefully remove PBS and discard the PFA waste.
    ii. Remove the chamber.
    iii. 2 × 5 min washing in PBS in a Coplin jar.
  c)Staining:
      i. Prepare first antibody solution.
    ii. Add 30 μl antibody solution to each well (see Supplementary File S1. Reagents and Solutions list).
    iii. Incubation: 60 min at 37°C or overnight (o.n.) at 4°C (see Supplementary File S1. Reagents and Solutions list).
    iv. 3 × 5 min washing in PBS in a Coplin jar.
    v. Prepare second antibody solution.
    vi. Add 30 μl antibody solution to each well.
    vii. Incubation: 30 min at 37°C.
    viii. 3 × 5 min washing in PBS in a Coplin jar.
    ix. Wash with _d_H_2_O in a Coplin jar.
    x. Drop the mounting medium on each well and carefully cover the slide with a cover slide.
**4|** Analysis of differentiation assay:
  a) Acquire two images per neurosphere, from the upper and lower part of the migration area without capturing the sphere core, by using a fluorescence microscope.
  b) Take a picture from exactly the same area of the nucleic acid staining (nuclei, blue) and O4 (oligodendrocytes, green), β-Tubulin III (neurons, green) or GFAP (astrocytes, red) staining, depending on the endpoint to measure.
  c) Save images with the respective experiment number.
  d) Install/open ImageJ.
  e) Count nuclei in a single-color image in ImageJ by processing the “binary watershed” function which separates merged nuclei and “analyzing particles” function.
  f) Overlay fluorescence pictures in an 8-bit format by merging the nuclei channel with O4, β-Tubulin III, or GFAP channel and save merged images in the RGB format.
  g) Subsequently, count the quantity of O4+ and β-Tubulin III + cells manually and normalize them by the number of nuclei.

##### P3.2. Migration of Neurospheres

**FIGURE 4:**
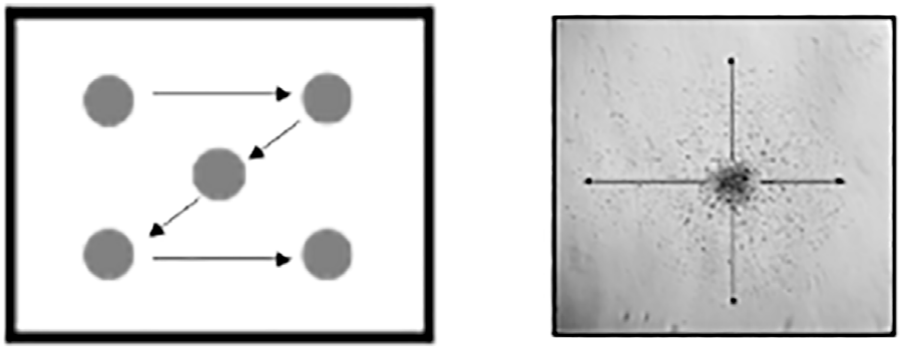
Schematic representation of the direction to follow while taking pictures for migration (left) and of the four distances to measure per sphere in the migration assay (right).

**1|** Brightfield pictures of the migration area:
  a) Take a brightfield image of each sphere on day 3 under differentiation conditions (see 3.1. Differentiation assay).
  b) Take a picture of the chamber number and one of each sphere in it following a ‘Z’ direction (see Figure 4).
  c) Save all pictures in a folder with the experiment identification number.
**2|** Analysis of cell migration in ImageJ:
  a) Install/open ImageJ.
  b) Open the images of the experiment to be analyzed.
  c) Click “Analyze”—“Set Scale”. Set scale from pixels to µm according to your microscope’s camera.
  d) Measure with the tool “straight line” the distance from the sphere core until the furthest migrated cell.
  e) Measure this distance four times in right-angled directions of the migration area.
  f) Calculate the mean of the four measurements.
  g) For each condition calculate the mean of the five spheres.

##### P3.3. Viability Assay (CellTiter Blue, CTB Assay)

For migration or differentiation assays:

1| Add DMSO to a final concentration of 10% 30 min before the addition of CTB-reagent to the lysis control well.
2| Mix CTB-reagent with N2 Media 1:3.
3| Discard 200 μl medium from slide chambers so that 300 μl medium remains.
4| Add the prepared CTB dilution 1:4 to each chamber (300 μl media in chamber + 100 μl dilution).
5| Incubate for 2 h at 37°C and 5% CO_2_.
6| Pipette 2 × 100 μl out of the 8-chamber slide into a 96 well plate (two replicates for each chamber).
7| Fixation steps can be performed now (see 3.1. Differentiation assay)
8| Measure in a fluorescence plate reader at 540 Ex/590 Em.

For proliferation assay:

1| Add DMSO to a final concentration of 10% 45 min before the addition of CTB-reagent to the lysis control wells.
2| Dilute CTB reagent 1:3 in B27 (without growth factors) media.
3| Add 33 μl to each well and incubate at 37°C and 5% CO_2_ for 2 h.

##### P3.4. Proliferation Assay

**1|** Preparation for neurosphere plating:
  a) Coat 96-well plates (clear, U-bottom) with 25 μl Poly-HEMA.
  b) 2–3 days earlier to the experimental plating day, chop rabbit neurospheres into 0.2-mm sized spheres.
  c) Prewarm B27 media with and without growth factors at 37°C.
  d) Prepare all desired treatment and control solutions and add 100 μl to each well of a 96-well plate previously coated with Poly-HEMA.
  e) Prepare solutions for four replicates per condition.
  f) Fill the surrounding wells with 100 μl sterile _d_H_2_O.
2| Plating of spheres:
  a) Sort the desired number of spheres (0.3 mm) and place them into a new Petri dish (60 mm) previously filled with 5 ml B27 media without growth factors (37°C).
  b) From the sorted spheres, transfer one sphere in 1.5 μl media into a well of a 96-well plate (U-bottom). Change the tip between different conditions.
  c) Take a picture of every sphere under the brightfield microscope using the camera on day 0 and 7 consecutive days at the same hour of the day.
  d) Incubate plate at 37°C, 5% CO_2_ for 7 days.
  e) Feed every 2 or 3 days the spheres by removing half of the media (50 μl) and adding fresh treatment and control solutions (prewarmed to 37°C).
  f) On day 7 a CellTiter Blue (CTB) assay for viability testing can be performed.
3| Analysis of the area increase in ImageJ.
  a) Install/open ImageJ.
  b) Open the images of the experiment to be analyzed.
  c) Measure the two diameters of the sphere and calculate the mean.
  d) Calculate the slope for the diameter increase and take the mean of 4 replicates per condition.

### Protocol 4. Behavioral Ontogeny

Alterations of behavior ontogeny are important neurodevelopmental adverse outcomes that can only be evaluated *in vivo*. In this protocol, the procedures needed to evaluate early behaviors of New Zealand rabbits at PND1 are presented together with the scoring system established to measure them. These procedures are based on the previous methodology described by Derrick et al. (2004).

#### P4 Materials and Equipment

- Data collection sheet (see Supplementary File S2).
- Prepare the observation area: put on a table the heating bed/electric blanket (50 × 50 cm approximately), switch it on at the lowest level, and cover it with an underpad. Draw a 15 cm line on the center of the underpad.
- Timer.
- Plastic Pasteur pipette loaded with warm puppy milk replacement formula (enriched with colostrum and omega 3).
- Ethanol-soaked gauze. Soak it again before each test.
- Optional: Recording setting (tripod and camera).

#### P4 Methods

This method can be evaluated in real-time if it is performed by two experimenters. In case only one experimenter is performing it, it needs to be recorded and evaluated afterward. The protocol consists of 12 tests scored with a scale from 0 to 3 or 0 to 4 (0: worst—3 or 4: best; see Table 1 for the detailed scoring system). In case the whole protocol is recorded, each animal should be recorded for 1 min. This one 1 min observation includes the evaluation of:

**TABLE 1:** Scoring system for behavioral ontogeny tests.

Endpoint	Score
Posture	0: lays supine
	1: lays on the side
	2: cannot maintain prone position, wobbly
	3: prone position with legs coiled
Righting reflex	Number of times the animal turns
Tone	0: no increase in tone
	1: slight increase in tone when the limb is moved
	2: marked increase in tone but the limb is easily flexed
	3: increase in tone, passive movement difficult
	4: limb rigid in flexion or extension
Circular motion	0: no movement
	1: slight movement; slight jump
	2: good range of motion; maintains for 1 or 2 steps; occasional jump
	3: entire range of motion; at least 3 steps; rapid jumps
Hind limb locomotion	0: no movement
	1: slight movement
	2: distinct movement
	3: rapid movement
Intensity	0: no movement
	1: slight activity
	2: distinct forceful movements
	3: rapid forceful movements
Duration	0: no movement
	1: activity <20 s
	2: activity 20–40 s
	3: activity >40 s
Lineal movement	Number of times crosses the perpendicular line
Fore-hind paw distance	Measurement (cm)
Sucking and swallowing	0: no movement of jaw; all milk dribbles out
	1: some movement of jaw; most of milk dribbles out
	2: definite suck and swallow; some milk in nose
	3: good suck and swallow; no milk in nose
Head turning	0: no movement
	1: slight occasional movement of the head
	2: distinct movement of the head
	3: rapid forceful movement of the head and body
Olfaction	0: no
	1: subtle
	2: low response
	3: correct
Olfaction time	Latency time (seconds)

General motor skills, tone, reflexes, and olfactory sensitivity scores grading, following previous methodology described by Derrick et al. (2004).

1| Place a PND1 New Zealand rabbit at the center of the observation area (optional: start video recording) and start the animal observation.

Test 1. Posture: place the animal in the observation area and evaluate the posture.

Test 2. Righting reflex: the pup will be forced to lay in supine position, and the number of times it manages to adopt the prone position in 10 trials will be recorded. Write down if it is an early or a late response since the expected behavior in controls is an immediate response.

Test 3. Tone: evaluate the muscle tone of the fore and hind limbs (right and left) by evaluating the resistance degree of the limb to passive flexo-extension. The less resistance the better score.

Test 4. Circular movement: it is the main movement performed by newborns (stronger forelimbs than hind limbs). Count the number of complete circular movements the pups perform. Simultaneously evaluate the jumps/hops associated with those circular movements (the faster and more frequent the better) for 1 min.

Test 5. Hind limbs locomotion: analyze the quantity and quality of the spontaneous head and limb movements associated with wandering.

Test 6. Intensity of the movements: non-spontaneous as well as almost spasmodic or very fast and energetic movements.

Test 7. Duration of movements: duration of continuous activity - not resting (within a min).

Test 8. Lineal movement. Place the animal perpendicular and 15 cm far from the line. Evaluate the number of times the pups cross the perpendicular line while walking a straight distance of 15 cm within a min.

Test 9. Shortest distance between fore and hind limbs: five measurements (in cm) of the distance between hind limbs and forelimbs. Consider only when walking straight. Write the mean down.

2| After this minute of evaluation, the following three tests are performed:

Test 10. Sucking and swallowing: place the plastic Pasteur pipette loaded with milk in the mouth of the pup. Evaluate the sucking reflex, milk spill, presence of milk in the nasal orifices as well as the overall head and body movement.

Test 11. Head movements: evaluate the head movements associated with the suction reflex when administering the milk in test 10.

Test 12. Olfactory test: soak a gauze in medical degree ethanol and bring it close to the nose of the pup without touching it. The Control group should have a quick aversive response such as moving the head away from the ethanol. Evaluate a null, mild, moderate or intense response, as well as the latency time in seconds.

3| Optional: Off-line analysis of the evaluation.

4| Fill in the data collection sheets.

### Protocol 5. Brain Sample Collection

This protocol describes the obtaining of brain samples at PND1 to be further processed in protocols 7, 8, and 9. For obtaining brain samples for protocol 6, please see protocol 6. For obtaining brain samples for protocols 13 and 14, please see protocol 12.

#### P5 Materials and Equipment

- 0.9% saline solution.
- 10% formalin.
- Sucrose 30% (see Supplementary File S1. Reagents and Solutions list).
- Plastic bags for freezing previously labeled with a different number for each brain.
- Dry ice.
- Scissors.
- Tweezers.
- Scale.

#### P5 Methods

1| Obtain the brain and record the weight.
2| Wash the brain with saline solution.
3| Fix the brain in 10% formalin for 24 h.
4| Immerse the whole brain in sucrose 30% for 48 h.
5| Collect the brain and place it in a labeled hermetic plastic bag for freezing.
6| Store samples at −80°C.

### Protocol 6. Golgi Staining Protocol

Protocols 6, 7, 8, and 9 describe the steps to perform neuropathological evaluations through Immuno-/stainings in brain slices of PND1 animals to evaluate adverse effects in neurons, astrocytes, microglia, and oligodendrocytes, respectively.

#### P6 Materials and Equipment

- FD Rapid GolgiStain kit (FD Neurotechnologies Inc.).
- Sterile 50 ml tubes.
- Double _d_H_2_O.
- Paintbrush.
- Slides.
- Vibratome.
- Epifluorescence microscope.

#### P6 Methods

##### P6.1. Obtaining Brain Tissue and Golgi Solution Incubations

1| Sacrifice the neonate by decapitation at PND1.
2| Obtain the brain and cut it into three different parts:
  a)Right hemisphere
  b)Left hemisphere
  c)Cerebellum and medulla
3| Wash the tissue with double _d_H_2_O to remove the blood.
4| Immerse the tissue in the supernatant A + B Solution (It is important to use the top part of the solution that is free of precipitate).
5| After 24 h renew solution A + B.
6| Incubate in A + B solution for 2 weeks at RT and in darkness. Gently swirl side to side for a few seconds twice a week.
7| After 2 weeks, transfer the tissue into solution C and store at RT in darkness for 3–7 days (ideally 5 days).
8| Replace the solution C once after 24 h (on the next day of the replacement).
9| Cut 100 µm sections with a vibratome (speed 5, amplitude 5), using Solution C or PBS as the medium.
10| Collect the sections with a paintbrush and mount them on adhesion or gelatin-coated slides.
11| Dry at RT in darkness, 2–3 days (max.).

Note: use at least 5 ml of the impregnation solution (solutions A, B, or C) for each cubic cm of tissue processed. It should be noted that using a lower volume of impregnation solution may decrease the sensitivity and reliability of staining.

##### P6.2. Staining Protocol

1| Rinse sections in double _d_H_2_O for 4 min (2 times).
2| Incubate with the stock solution for 10 min (prepare just before use as follows):
  a) 1 part of Solution D.
  b) 1 part of Solution E.
  c) 2 parts of double _d_H_2_O.
3| Rinse with double _d_H_2_O for 5 min (4 times).
4| Contrast: incubate for 6 min with Cresyl violet
5| Dehydration:
  a) 4 min, 50% ethanol.
  b) 4 min, 75% ethanol.
  c) 4 min, 95% ethanol,
  d) 4 min, ethanol absolute (4 times).
6| Xylene for 4 min (3 times).
7| Cover slip with Permount and dry at RT protected from light o.n.
8| Acquire images under 40x objective magnification in an AF6000 epifluorescence microscope.
9| Analysis of the images. Here we present an example evaluating pyramidal neurons; however, other neuronal types can be included depending on the interest of the study. Five pyramidal neurons from the area of interest from each brain hemisphere (10 neurons per animal) that fulfill the inclusion criteria are randomly selected.

Inclusion criteria: pyramidal neurons within layers II and III, and complete filling of the dendritic tree, especially for the basal dendrites, as evidenced by well-defined endings.

10| Measure the following parameters from each neuron using ImageJ software:
  a) Area of the soma (obtained by manual delineation of the shape of the neuronal soma in a 2D image).
  b) Number of basal dendrites (obtained by manual counting).
  c) Total basal dendritic length (obtained after performing manual delineation of the length of each basal dendrite and then calculating the addition of all lengths from all basal dendritic branches).
  d) Basal dendritic complexity, which includes the evaluation of the number of basal dendritic intersections and the number of each basal dendritic branches. The basal dendritic complexity is evaluated by using the Sholl technique, as previously described (Sholl, 1953). Sholl rings are placed concentrically in 10 µm increasing intervals centered on the soma. For the basal dendritic intersections, the number of intersections that dendritic branches have per each Sholl ring and the addition of all of them are recorded. For the number of each basal dendritic branch, each basal dendritic branch is divided into primary, secondary, tertiary, quaternary, quinary, and senary dendrites. Primary dendrites are considered those dendrites that are originated from the soma; secondary dendrites those that are derived from the primary dendrites, and so on, up to the senary dendrites, corresponding to those derived from the quinary dendrites.

### Protocol 7. Astrocyte IHC

#### P7 Materials and equipment

-Anti-GFAP (GA-5): NBP2-29415- 20 µg (Bio-Techne).
-Goat anti-Mouse IgG secondary antibody.
-PBS.
-PBST 0.3%.
-Citrate buffer (pH 6).
-IHC blocking solution (see Supplementary File S1. Reagents and Solutions list).
-Mounting media.
-Humid chamber.
-Slides.
-Hydrophobic pen.
-Paintbrush.
-Confocal scanning laser microscope.

#### P7 Methods

1| Obtain and store PND1 brains, as detailed in PROTOCOL 5.
2| Acquire consecutive 40 μm sections by cryotomy.
3| Select the sections containing the area of interest with the help of a rabbit brain atlas (Muñoz-Moreno et al., 2013).
4| Use a slide per animal with three consecutive cuts in each slide and delimit them with a hydrophobic pen.
5| Process the slides for heat-induced-epitope-retrieval (HIER) in citrate buffer (pH 6) for 3 min (at 90°C in a Coplin jar inside a double-boiler).
6| Permeabilize tissue with Triton X-100 0.3% in PBS for 30 min at RT in a humid chamber.
7| Block the samples by incubating slides with 1% BSA and 5% goat serum for 1 h at RT.
8| Incubate tissue sections with 1:400 anti-GFAP at 4°C o.n.
9| Wash 3 times with Triton X-100 0.3% in PBS for 5 min.
10| Incubate with goat anti-mouse IgG and 1:1000 nucleic acid stain for 1 h at RT.
11| Wash once with Triton X-100 0.3% in PBS for 5 min at RT.
12| Wash twice with PBS for 5 min at RT.
13| Rinse in _d_H_2_O.
14| Add mounting media and store at 4°C o.n.
15| Seal with nail polish and store at -20°C.
16| When slides are dry, observe them with confocal scanning laser microscopy.
17| Acquire images with a 63x/1.40 oil immersion differential interference contrast (DIC) objective. Quantify images by counting GFAP + cells/mm^2^.

### Protocol 8. Microglia IHC

#### P8 Materials and Equipment.

- Primary antibody: biotinylated *Lycopersicon esculentum* tomato lectin (VectorLabs #B-1175).
- Cy3-conjugated streptavidin.
- PBS.
- PBST 0.3%.
- Nucleic acid stain.
- IHC blocking solution (see Supplementary File S1. Reagents and Solutions list).
- Slides.
- Cryomicrotome.
- Paintbrush.
- Mounting media.
- Hydrophobic pen.
- Nail polish.
- Confocal scanning laser microscope.

#### P8 Methods

1| Obtain and store PND1 brains, as detailed in PROTOCOL 5.
2| Obtain with the cryomicrotome 40 µm cuts covered with poli-L-lysine.
3| Select the sections containing the area of interest with the help of a rabbit brain atlas (Muñoz-Moreno et al., 2013).
4| Use a slide per animal with three consecutive cuts in each slide.

In all steps, add 50–100 μl/cut, except for the antibody incubation steps, where 50 μl/cut has to be added but the excess solution has to be removed with the vacuum.

5| Thaw the slides with the samples and leave them at RT to dry for 10 min.
6| Delimit the slide using a hydrophobic pen and wait until it is completely dry.
7| Permeabilize with PBST 0.3% with 0.5% BSA at RT for 1 h.
8| Incubate with the primary antibody biotinylated *Lycopersicon esculentum* tomato lectin at RT for 60 min.
9| Wash the samples with PBST 0.3%.
**10|** Incubate with conjugated Cy3-conjugated Streptavidin for 30 min at RT with 1% nucleic acid stain.
11| Wash the samples with PBST for 5 min at RT.
12| Wash the samples two times with PBS for 5 min at RT.
13| Immerse the samples in _d_H_2_O one time.
14| Add mounting media (80 μl/slide) and remove the excess.
15| Keep in the fridge until the next day.
16| Then, 24 h later, seal with nail polish and store at -20°C.
17| Observe with confocal scanning laser microscopy.
18| Images are taken under 40x objective magnification with 10 steps of 1 µm in the Z-stack. The total number of stained cell nuclei and the number of cells with positive fluorescent staining around the nucleus are counted using ImageJ software. The number of positive cells/mm^2^ is then calculated.

### Protocol 9. Oligodendrocyte IHC

#### P9 Materials and equipment.

- Slides.
- Paintbrush.
- PBS.
- PBST 0.3%.
- Nucleic acid stain.
- IHC blocking solution (see Supplementary File S1. Reagents and Solutions list).
- Primary antibody Mouse IgM anti-O4 (Merck Millipore #MAB345).
- Secondary antibody goat anti-mouse IgM.
- dH_2_O.
- Mounting media.
- Nail polish.
- Cryostat.
- Hydrophobic pen.
- Confocal scanning laser microscope.

#### P9 Methods

1| Obtain and store PND1 brains, as detailed in PROTOCOL 5.
2| Obtain with the cryostat 40 µm cuts. Use a slide per animal with three consecutive cuts in each of them.

In all steps, add 50–100 μl/cut, except for the antibody incubation steps, where 50 μl/cut has to be added but the excess solution has to be removed with the vacuum.

3**|** Thaw the slides with the samples and leave them at RT to dry for 10 min.
4| Delimit the slide using a hydrophobic pen and wait until it is completely dry.
5| Wash the samples two times with PBS for 5 min at RT.
6| Permeabilize with PBST 0.3% at RT for 20 min.
7| Incubate with the IHC blocking solution in a humidity chamber at RT for 1 h.
8| Incubate with the primary antibody Mouse IgM anti-O4 (#MAB345) o.n. at 4°C (ensure more than 14 h incubation).
9| Leave the sample at RT for 60 min.
10| Wash the samples three times with PBST 0.3% for 5 min at RT.
11| Incubate for 1 h with the secondary antibody goat anti-mouse IgM at RT with 1% nucleic acid stain.
12| Wash the samples with PBST 0.3% for 5 min at RT.
13| Wash the samples with PBS for 5 min at RT.
14| Immerse the samples in _d_H_2_O one time.
15| Add mounting media (80 μl/slide) and remove the excess.
16| Keep in the fridge until the next day.
17| 24 h later, seal with nail polish and store at -20°C.
18| Observe with a confocal scanning laser microscope.
19| Images are taken under 40x objective magnification with 10 steps of 1 µm in the Z-stack. The total number of stained cell nuclei and the number of cells with positive fluorescent staining around the nucleus are counted using ImageJ software. The number of positive cells/mm^2^ is then calculated.

### Protocol 10. Skinner Box

Operant conditioning behavior can be studied using a Skinner box adapted to rabbit. In this test, the response of the animals to the environment when they receive food reinforcements is evaluated and can be used as a measure of learning. According to OECD TG 426, learning and memory tests should be performed in adolescents as well as in young adult animals. Here, we present a protocol for performing the Skinner box test in young adults on PND70, which is adapted from Zworykina et al. (1997). Further evaluations are needed to establish if this protocol can directly be used during the adolescence period, if the protocol needs adaptations, or if another learning test should be used at that age since the TG allows the use of the same or different tests at these two stages of development.

#### P10 Materials and Equipment

- Data collection sheets.
- Neurological observation area: Skinner box for rabbits constructed as detailed in Leal-Campanario et al., (box for operant conditioning and instrumental learning for rabbits, 2012. Inscription number in Spain: P2001231369). Briefly, the operant box (780 × 595 × 985 mm) has three aluminum lateral walls and the central lateral wall is made of methacrylate. The floor is a 590 × 815 mm multi-perforated PVC plate that allows the free movement of the animal. It also includes a tray with sawdust bedding to collect the feces. The lever (130 × 100 mm), made of aluminum, is located on one of the side walls, 30 mm above the floor level, and protrudes 67.5 mm toward the inside of the box. In addition, it has a recovery mechanism to return to its starting position. The lever is connected to a control panel. The food dispenser is located outside of the box and connected to the feeder. The food dispenser is connected to the control panel and receives a signal when the lever has been hit, allowing a pellet to fall into the feeder. The diameter of the feeder is 60 mm and it is located 10 mm above the floor level.

#### P10 Methods

##### P10.1. Reduction of Food Intake and Body Weight Control

**1|** Weighing the animals daily. The goal is to achieve a 15% reduction of the initial weight before starting the test.
**2|** Proceed with the intake reduction protocol:
  a) Monday to Tuesday of the week prior to the start of the test: 80 g of food/day + hay.
  b) Afterward and during the Skinner test: 20 g/day + hay.
  c) On weekends: 20 g/day + hay.

##### P10.2. Skinner Box Test

Drive the animals to the experimental room 30 min before the test. The experimental room has to be dimly lit and present standard conditions. Clean the apparatus with 70% ethanol.

1| During the first week (PND50-59): habituation and training.
a) First day of exploration: habituation for 10 min. Record global animal behavior (freezing, defecation and urination, exploration, approach to the feeder and lever, rearing and grooming).
b) Second day of exploration (training 1): 10 min.
  i. Reinforce the feeder. Put food in the feeder whenever the animal explores the feeder, after exploring the rest of the box.
  ii. Register the number of reinforcements administered in the first 5 min and the last 5 min.
  iii. Register in observations the global animal behavior.
c) Third day of exploration (training 2): 10 min.
  i. Reinforce the feeder and the lever. Put food in the feeder and the lever whenever the animal explores the feeder and the lever, after exploring the rest of the box.
  ii. Register the number of reinforcements administered in the first 5 min and the last 5 min.
  iii. Register in observations the global animal behavior.
c) Fourth day of exploration (training 3): 10 min.
  i. Reinforce the lever. During the first 5 min: feed the lever and then reward the animal with food in the feeder. During the last 5 min: reinforce the lever with only smell and then reward the animal with food in the feeder.
  ii. Register the number of reinforcements administered in the first 5 min and the last 5 min.
  iii. Register in observations: global animal behavior and whether or not it takes a long time to search for food from the feeder.
c) Fifth day of exploration (training 4): 10 min.
  i. Reinforce the lever with smell and then reward the animal with food in the feeder (the first stimulus can be putting food in the lever).
  ii. Register the number of reinforcements administered in the lever in the first 5 min and the last 5 min.
  iii. Register in observations: global animal behavior and whether it takes a long time to search for food from the feeder.
2| During the second week (PND60-69): fixed reason 1 (FR1: one lever–one piece of food).
a) First-day session FR1: 10 min.
  i. Only administer food when the animal presses the lever.
  ii. At the beginning of the session you can administer a reinforcement in the lever. If the animal does not remember, you can give other level reinforcements throughout the examination (maximum 2–3).
  iii. Register the number of levers hit correctly during the first 5 min and the 5 last min.
  iv. Register in observations: global animal behavior and whether it takes a long time to search for food from the feeder once it has hit the lever.
b) Second-day session FR1: 10 min.
c) Third-day session FR1: 10 min.
d) Fourth-day session FR1: 10 min.
  i. Only administer food when the animal presses the lever.
  ii. At the beginning of the session you can administer reinforcement in the lever. You should not give any more reinforcements.
  iii. Register the number of levers hit correctly during the first 5 min and the 5 last min.
  iv. Register in observations: global animal behavior and whether it takes a long time to search for food from the feeder once it has hit the lever.
e) Fifth-day session FR1: 10 min.
  i. Only administer food when the animal presses the lever.
  ii. You should not give reinforcements.
  iii. Register the number of levers hit correctly during the first 5 min and the 5 last min.
  iv. Register in observations: global animal behavior and whether it takes a long time to search for food from the feeder once it has hit the lever.
3| Complete the data collection sheets.
4| Weigh animals and offer food *ad libitum*.

### Protocol 11. Open-Field Test and Object Recognition Test

These two common behavioral assays are used to evaluate motor activity in the open field test, and learning and memory, combined with sensory function (olfactory) in the object recognition test. This protocol is established to perform the open field behavioral test, and the phase A of the object recognition test one after the other with the same animal on PND70. Once all animals have performed both tests, phase B of the object recognition test starts following the same animal order. OECD TG 426 requires motor and sensory functions to be examined in detail at least once for the adolescent period and once during the young adult period. The open-field test can probably be also used during the adolescence period, but further studies checking the adjustments needed for this developmental stage are required.

#### P11 Materials and Equipment

-Data collection sheets.
-Two apples and one orange.
-Self-made open field box (140 × 140 cm; surrounded by a 40 cm height wall).
i.Preparation of the open field box: avoid light reflection on the floor by keeping the room dimly lit.
ii.Preparation for the object recognition test: two specimen collection containers with two identical fruits (familiar object) and a third specimen collection container with a different fruit (novel object). In this case, two apples as familiar objects and one orange as novel objects were used. They were sliced and put in the container. The lids must have some holes to release the smells.
-Video Tracking Software.
-Ethanol-soaked gauze. Soak it again before each test.
-Recording setting (tripod and camera).

#### P11 Methods

##### P11.1. Open-Field Test

Drive the animals to the experimental room 30 min before the test. The experimental room has to be dimly lit and present standard conditions. Clean the open field box with 70% ethanol.

1| Open the video tracking software and start a new experiment.
2| Identify the animal and name the file (use the same naming once the acquisition is finished).
3| Define acquisition settings and save them as a default:
  a)Define the observation area: adjust the acquisition limits setting them to 140 × 140 cm.
  b)Adjust brightness and contrast if necessary.
4| Start the test: start recording:
  a) Put the cloth inside the open field and start filming (consider if the video tracking software needs some recording of the open field without the animal).
  b) Immediately after, pick the subject, cover it with a cloth, place the animal in the starting point and remove the covering cloth. The starting point is defined as a limited field of about 1/5 of the whole observation area, preferably the opposite site to the corridor where the camera is set.
  c) The observer must leave the room.
  d) After 10 min of recording, the observer enters the room and picks the subject up. Then, and not before, the recording is stopped. Save the file using the same naming.

##### P11.2. Object Recognition Test: Phase A or B

1| Open the video tracking software and start a new experiment.
2| Identify the animal and name the file (use the same naming once the acquisition is finished. Indicate whether it is phase A or B).
3| Define acquisition settings and save them as a default
  a) Define the observation area: adjust the acquisition limits setting them to 140 × 140 cm.
  b) Adjust brightness and contrast if necessary.
4| Start the test: start recording
a) Two separated familiar objects are placed in the center of the observation area.
  i. Phase A evaluation: two identical familiar objects are placed in the area.
  ii. The familiar object is a specimen collection container that has a drilled lid to enable recognition and is filled with apple slices.
  iii. Leave an inter-trial time of 30 min between phase A and phase B
  iv. Phase B: a familiar object is replaced by a novel one. In this case, a new drilled-lid specimen container is filled with orange slices.
  v. There is no need to wash the fruit rigorously or control the size of the slices.
b) The recording must start at least 1 s before bringing the subject in.
c) Immediately after the animal is placed at the starting point, the observer must leave the room during the 5-min recording.
d) After 5 min recording, the observer enters the room and picks the subject up. Then, and not before, the recording is stopped. Save the file using the same naming.

Every subject is studied individually to avoid unwanted smell recognition.

5| Fill in the data collection sheets.

### Protocol 12. Brain Sample Collection

This protocol describes the obtaining of brain samples at PND70 to be further processed in protocols 13 and 14. For obtaining brain samples for protocol 6, please see protocol 6. For the obtaining of brain samples for protocols 7, 8 and 9, please see protocol 5.

#### P12 Materials and Equipment

- 0.9% saline solution.
- Paraformaldehyde 4%.
- Sucrose 30% (see Supplementary File S1. Reagents and Solutions list).
- Plastic bags for freezing labeled with a different number for each brain.
- Dry ice.
- A 100 ml beaker filled with 2-methyl butane (cold).
- Guillotine.
- Scissors.
- Tweezers.
- Infusion pump.
- Needle 18G.
- Scale.

#### P12 Methods

1|Perfuse the animal with 0.9% saline and paraformaldehyde 4%.
2| Obtain the brain and record the weight.
3| Immerse the whole brain for 24 h in paraformaldehyde 4%.
4| Immerse the whole brain in sucrose 30% for 48 h.
5| Cut the brain as needed depending on the area of interest (it is recommended to cut it at least in three parts to ease the cutting step with the cryotome).
6| Incubate the brain samples in 2-methyl butane (placed in dry ice) for 1 min.
7| Collect the brain samples and place them in a labeled hermetic plastic bag for freezing.
8| Store samples at -80°C.

### Protocol 13. Dendritic Spines IHC

Protocols 13 and 14 describe the steps to perform neuropathological evaluations through Immuno-/stainings in brain slices of PND70 animals to evaluate adverse effects in dendritic spines or perineuronal nets, respectively.

#### P13 Materials and Equipment

- Gene Gun System.
- Tubing for the Gene Gun System.
- Dil Stain.
- Cryomicrotome.
- Methylene chloride.
- Tungsten particles (1.7 mm diameter).
- Slides.
- _d_H_2_O.
- Nitrogen flow gas.
- Membrane filter of 3 μm pore size and 8 × 10 pores/cm^2^.
- PBS.
- DAPI.
- Mounting media.
- Confocal microscope with a 63x oil-immersion objective.

#### P13 Methods

1| Obtain the brain at PND70, as detailed in PROTOCOL 12.
2| Acquire 150 μm coronal sections by cryotomy.
3| Prepare a suspension containing 3 mg of Dil dissolved in 100 μl of methylene chloride mixed with 50 mg of tungsten particles.
4| Spread the suspension on a slide to air-dry.
5| Resuspend the mixture in 3.5 ml _d_H_2_O and sonicate it.
6| Drawn the mixture into a Tefzel tubing and remove it to allow the tube to dry under nitrogen flow gas for 5 min.
7| Cut the tube into 13 mm pieces to be used as gene gun cartridges.
8| Deliver particles to the area of interest using a modification of the gun to enhance accuracy by restricting the target area.
9| Deliver Dil-coated particles in the area of interest shooting over 150 μm coronal sections at 80 ψ through a membrane filter of 3 μm pore size and 8 × 10 pores/cm^2^.
10| Store sections in PBS at RT for 3 h protected from light.
11| Incubate with DAPI and use mounting media to be analyzed.
12| Image Dil-labeled pyramidal neurons from the area of interest using a confocal microscope with a 63x oil-immersion objective.
  a) Held constant throughout the study the pinhole size (1 AU) and frame averaging (four frames per z-step).
  b) Take confocal z-stacks with a digital zoom of 5, a z-step of 0.5 μm, and at 1,024 × 1,024 pixel resolution, yielding an image with pixel dimensions of 49.25 × 49.25 μm.
13| Select two or three basal dendrites of various neurons for the analysis of spine density.
  a) Select segments with no overlap with other branches that would block visualization of spines.
  b) Select segments either “parallel” to or “at acute angles” relative to the coronal surface of the section to avoid ambiguous identification of spines.
  c) Select spines arising from the lateral surfaces of the dendrites and dendritic segments of basal dendrites 45 μm away from the cell body.

### Protocol 14. Perineuronal Nets IHC

#### P14 Materials and equipment.

- PBS.
- PBST 0.3%.
- Nucleic acid stain.
- Cryomicrotome.
- IHC blocking solution (see Supplementary File S1. Reagents and Solutions list).
- Primary antibody: lectin from *Wisteria floribunda* (Sigma #L1516; 1:20).
- Secondary antibody: streptavidin, Alexa Fluor 488 conjugate (1:2000).
- Slides.
- Paintbrush.
- Hydrophobic pen.
- Mounting medium.
- Confocal scanning laser microscope.

#### P14 Methods

1| Obtain the brain at PND70 as detailed in PROTOCOL 12.
2| Acquire 20 μm sections by cryotomy and include three sections per slide.
3| Dry sections at 37°C for 10 min and store at -20°C.
4| Thaw sections and dry at 37°C.
5| Delimit the slide using a hydrophobic pen and wait until it is completely dry.
6| Hydrate with PBS for 10 min.
7| Permeabilize with PBST 0.3%.
8| Block the samples with IHC blocking solution at RT for 60 min.
9| Incubate o.n. with the primary antibody at 4°C (400 μl/slide).
10| Leave samples at RT for 1 h.
11| Wash the samples two times with PBST 0.3% (from now on, protect the samples from light).
12| Incubate with the secondary antibody at RT (400 μl/slide) for 90 min.
13| Wash the samples four times with PBST 0.3%.
14| Wash the samples with PBS for 10 min.
15| Incubate with nucleic acid stain 1/1000 with PBS for 20 min.
16| Wash the samples with PBS for 10 min.
17| Wash the samples with PBS for 10 min.
18| Dry the samples and add mounting medium, with 60 μl/slide covering the tissue.
19| Observe with a confocal scanning laser microscope
20| Images are taken under 40x objective magnification with 20 steps of 1 µm in the Z-stack.
21| Analyze perineuronal nets by quantifying the average density of immunolabeling (contacts/µm^2^) in the region of interest.

A summary of critical parameters and troubleshooting of protocols described in this section is presented in Table 2.

**TABLE 2:** General summary of critical parameters and troubleshooting.

Protocol	Problem	Possible reason	Solution
2	Rabbit neurospheres do not form/proliferate	Rabbit NPC need to be cultured in a relatively high density to form neurospheres	After thawing neurospheres, use small 60 mm Petri dishes. As soon as they have a certain size (ca. 11 days in proliferation media) chop or transfer the neurospheres to a 90 mm Petri dish
3	Too low oligodendrocyte differentiation	Neurospheres are kept too long out of the incubator	Do not plate longer than 30 min one 8-chamber slide
3	The migration area of different spheres overlaps or is too close to the chamber edges	Neurospheres were plated too close to the chamber edge or too close to each other	Place neurospheres like the dots of a dice in face number 5
3	The lysis of the neurospheres is not completed	Proliferating neurospheres need more time to lyse than differentiating neurospheres	Incubate proliferating neurospheres for 45 min and differentiated ones for 30 min in lysis control (10% DMSO)
3	Spheres easily detach from the slide surface	The fixation is not correct	Do not thaw PFA more than once
3	Neurospheres detach from the slide surface during the staining	Problems with pipetting during washing steps	Pipette gently and carefully during the washing steps
3	High background signal	Less washing steps than indicated	Wash as indicated
4	Some of the pups are very weak	—	Make sure the bed is warm and the milk preparation is warm
4	Difficulties with the simultaneous evaluation of the parameters	—	Conduct the test in pairs. If not, record the whole process
4	The animal gets out of the observation area	—	Get it back and continue the observation
6	Difficulties in finding neurons with well-defined endings	Brain sectioning problems	Manipulate carefully the tissue slices when performing the 100 µm cut sections with the vibratome
6	It takes a long time for image evaluation	A lot of parameters to be evaluated in the same neuron	Try to analyze one neuron for each experimental group each day you perform the analysis
7	Difficulties in the cryosectioning of samples	Temperature difference between the sample and the cryomicrotome	Put the samples stored at -80°C in the freezer (-20°C) o.n. to facilitate cryosectioning
7	Weak or absent fluorescence	Anti-GFAP antibodies do not reach the protein	Perform HIER (heat-induced epitope retrieval)
7	Detachment of the tissue slices	Incorrect manipulation of the tissue slices. Inappropriate coating of the slide	Manipulate carefully the tissue slices and liquids. Select slides for cryosectioning
7	Unspecific fluorescence in the IHC	Incorrect blocking step	Always include a negative control of each subject to define unspecific fluorescence
10	Stressed/hungry animals	Intake reduction	Try to perform the evaluation early in the morning and afterward give them the food indicated by the protocol
10	Some animals do not move inside of the Skinner box	They are scared	In the habituation and training week, try to stimulate them by doing noise in the lever and the feeder
11	Anomalous initial response in the open-field test and object recognition test	The animal is stressed	Manipulate the animal with kindness and try to stay silent while the test is running

## Results

The results summarized in this section include the control values obtained in several studies evaluating the adverse effects of a mild and chronic hypoxia-ischemia insult during gestation in one of the uterus horns by ligation of 40–50% of uteroplacental vessels on day 25 of pregnancy (Illa et al., 2013; Illa et al., 2017; Illa et al., 2018; Pla et al., 2021). The control values presented here are from GD30 fetuses in the contralateral control horn and are therefore obtained from control fetuses after a cesarean section and not by natural delivery.

*In vitro* results are presented as the mean of independent experiments (n = 5–25) with standard deviation (SD) and include at least five neurospheres per condition in each independent experiment. *In vivo* results are presented as mean values of controls with SD, but the mean is calculated in two different ways, considering the litter or the pup/hemisphere/cell as a statistical unit for later comparison.

*In vitro* results of the neurosphere assay (Protocol 3) are summarized in Table 3, where both solvent control values and specific positive control values for each endpoint are presented. Details on which positive control was used for each endpoint are given in Table 4. These results show the ability of rabbit neurospheres to perform several processes of neurogenesis and demonstrate that the different endpoints can be affected by exposure to known disturbing substances (positive controls) as described before (Barenys et al., 2021). Previous work has also provided evidence that the technique can detect alterations after exposure to a known developmental neurotoxicant like methylmercury chloride, while no significant alterations are observed after exposure to a negative control like saccharine (Barenys et al., 2021). From the experience generated, a quality threshold has been established for each endpoint, which is summarized in Table 4. If control results do not reach the minimum values indicated in this table, the experiment should be discarded, otherwise, the effects of the compounds could be overestimated.

**TABLE 3:** Results of PROTOCOL 3

Endpoint	Solvent control *mean ± SD* (n)	Positive control *mean ± SD* (n)
Oligodendrocyte differentiation (%)	6.2 ± 0.4 (24)	0.6 ± 0.1 (15)
Neuronal differentiation (%)	2.4 ± 0.4 (14)	0.4 ± 0.2 (8)
Migration distance (µm)	803.5 ± 46.4 (22)	238.5 ± 40.2 (8)
Proliferation (µm of diameter increase/day)	15.7 ± 3.6 (5)	-3.6 ± 1.2 (5)
Viability (RFU)	22406.0 ± 1670.2 (25)	2005 ± 299.4 (22)

**TABLE 4:** Threshold PROTOCOL 3

Endpoint	Endpoint-specific Positive control	Exclusion criteria in experiments with rabbit neurospheres
Oligodendrocyte differentiation	100 ng/ml BMP7	5 days diff: < 1.5% in solvent control
Neuron differentiation	10 ng/ml EGF	5 days diff: < 1.5% in solvent control; 3 days diff: < 1% in solvent control
Migration distance	10 µM PP2	<250 µm in solvent control
Viability	10% DMSO	<5700 RFU
Proliferation	B27 media without growth factors	<10 µm of diameter increase/day

*In vivo* results for protocols performed on PND1 are presented in Table 5: Behavioral ontogeny, Table 6: Golgi staining, and Table 7: Astrocytes, microglia and oligodendrocytes IHC. Afterward, results of protocols performed from PND50 are presented in Table 8 for Skinner box test, open field and object recognition test, and Table 9 for dendritic spines and perineuronal nets evaluation. In all these tables, the symbol # indicates results where variability is equal to or higher than the mean of controls for that particular way of calculation. This has been highlighted to allow the detection of measured endpoints with high variability in relation to the mean value, which in fact can render the endpoint as not useful to discriminate adverse effects.

**TABLE 5:** Results of PROTOCOL 4.

Endpoint (units)	Statistical unit: pup (*n* = 11) *Median* (*IQR*) *or mean ± SD*	Statistical unit: litter (*n* = 4) *Median* (*IQR*) *or mean ± SD*
Posture (score)	Mdn: 3.0 (0.0)	Mdn: 3.0 (0.0)
Righting reflex (n. of times)	M: 9.3 ± 1.4	M: 9.5 ± 0.8
Tone (score)	Mdn: 4.0 (0.0)	Mdn: 4.0 (0.0)
Circular motion (score)	Mdn: 3.0 (1.0)	Mdn: 2.8 (0.6)
Hind limb locomotion (score)	Mdn: 3.0 (1.0)	Mdn: 3.0 (0.4)
Intensity (score)	Mdn: 3.0 (0.0)	Mdn: 3.0 (0.0)
Duration (score)	Mdn: 3.0 (0.0)	Mdn: 3.0 (0.0)
Lineal movement (n. of times)	M: 2.9 ± 1.7	M: 2.9 ± 1.1
Fore-hind paw distance (cm)	M: 0.5 ± 1.2^#^	M: 0.6 ± 0.8^#^
Sucking and swallowing (score)	Mdn: 3.0 (0.5)	Mdn: 3.0 (0.6)
Head turning (score)	Mdn: 3.0 (0.0)	Mdn: 3.0 (0.1)
Olfaction (score)	Mdn: 3.0 (1.0)	Mdn: 2.3 (0.6)
Olfaction time (seconds)	M: 3.4 ± 2.6	M: 3.0 ± 1.6

The mean (M) ± SD is presented for righting reflex, lineal movement, fore-hind paw distance, and olfaction time; the median (Mdn) and (Interquartile Range (IQR)) are presented for the rest of the endpoints.

**TABLE 6:** Results of PROTOCOL 6

Endpoint	Statistical unit: neuron *mean ± SD* (*n* = 40)	Statistical unit: litter *mean ± SD* (*n* = 3)
Total length (µm)	676.2 ± 289.3	681.0 ± 94.2
Number of principal branches	7.2 ± 2.1	7.3 ± 0.6
Number of secondary branches	8.3 ± 3.2	8.2 ± 0.8
Number of tertiary branches	3.5 ± 2.6	3.3 ± 1.1
Number of quaternary branches	1.1 ± 1.5^#^	0.9 ± 0.6
Number of quinary branches	0.2 ± 0.6^#^	0.2 ± 0.2^#^
Number of senary branches	0.1 ± 0.6^#^	0.1 ± 0.1^#^
Number of quaternary, quinary, and senary branches	1.4 ± 2.2^#^	1.2 ± 0.9
Total branches	20.5 ± 7.6	20.0 ± 2.9
Area soma (µm^2^)	337.5 ± 79.5	322.9 ± 51.2

**TABLE 7:** Results of PROTOCOLS 7, 8, and 9

Endpoint	Statistical unit: pup *mean ± SD* (*n*)	Statistical unit: litter *mean ± SD* (*n*)
Astrocyte IHC (from protocol 7) (GFAP+ cells/mm^2^)	7.6 ± 3.0 (7)	8.7 ± 2.2 (4)
Microglia IHC (from protocol 8) (tomato lectin positive cells/mm^2^)	13.8 ± 8.1 (8)	12.4 ± 6.0 (4)
Oligodendrocyte IHC (from protocol 9) (O4+ cells/mm^2^)	50.4 ± 16.7 (8)	48.7 ± 12.4 (4)

**TABLE 8:** Results of PROTOCOLS 10 and 11.

Endpoint	Statistical unit: pup *mean ± SD* (*n*)	Statistical unit: litter *mean ± SD* (*n*)
Learning criteria (% of animals meeting the learning criterion)	77 ± 44 (13)	86 ± 22 (6)
Rate of acquisition (mean increase in lever hits between sessions)	1.4 ± 0.3 (13)	1.4 ± 0.3 (6)
Learning day	6.5 ± 2.5 (13)	6.4 ± 2.7 (6)
Of latency time (s)	122.0 ± 71.5 (13)	125.4 ± 83.6 (6)
Of total distance (cm)	3566.3 ± 2749.2 (13)	2575.0 ± 2840.7# (6)
Of time center (s)	31.2 ± 20.4 (13)	30.9 ± 15.6 (6)
Of time periphery (s)	490.3 ± 114.5 (13)	479.5 ± 126.8 (6)
ORT Discrimination Index (DI)	0.1 ± 0.3 (17)	0.1 ± 0.3 (8)

**TABLE 9:** Results of PROTOCOLS 13 and 14.

Endpoint	Statistical unit: hemisphere *mean ± SD* (*n*)	Statistical unit: litter *mean ± SD* (*n*)
Density of dendritic spines (number of spines/µm) from protocol 13	1.8 ± 0.2 (6)	1.8 ± 0.2 (4)
Perineuronal nets (contacts/µm^2^) from protocol 14	0.24 ± 0.05 (6)	0.25 ± 0.05 (4)

In the behavioral ontogeny evaluation (Table 5) the expected scores for control pups are, in general, the maximum possible scores of the scale, with few exceptions of pups receiving the next maximum score possible. This results in very high scores for almost all endpoints and relatively low standard deviations in the control group. There is only one endpoint showing a deviation bigger than the mean value itself, the endpoint “Fore-hind paw distance,” but this is an endpoint in which the treatment effect is expected to probably increase the mean value and not decrease it as in all other endpoints evaluated with a score. Due to this difference in the dynamic range of the endpoint, it is still considered a valuable and informative endpoint of the battery, worth being included in it, despite the variation in the controls.

Neuronal evaluation with Golgi staining was performed in the frontal cortex of PND1 rabbits, as detailed by Pla et al. (2021). Neurons presented mainly principal and secondary branches in a similar proportion, while tertiary branches were less than half of the previous ones. At this developing time and in this area of analysis, there were almost no quaternary, quinary and senary branches. In fact, the number of the three latest ones was so low that small variations resulted in a high relative variability compared to the mean value. Because of that, if reductions in the number of branches are expected, it is suggested to evaluate the number of branches above tertiary altogether to improve the sensitivity and the dynamic range of the endpoint, since these measurements are not difficult to be performed and can still be informative.

IHCs of astrocytes, microglia, and oligodendrocytes were evaluated in the corpus callosum genu area and the results expressed as GFAP + cells/mm^2^, tomato lectin positive cells/mm^2^ and O4+ cells/mm^2^. The most abundant cells in this area were oligodendrocytes but the density of cells in these three endpoints was enough to allow a positive and negative dynamic range in them. Their variabilities were low in all cases except for microglial density when the statistical unit was the pup. In this case, variability was higher than 50% of the mean control value but still lower than the mean.

In the Skinner box, the learning criterion was considered to be met when the animal pressed the lever and went directly toward the food dispenser to obtain the reward at least three times in one session. 86% of controls (statistical unit = litter) reached this learning criterion and they did it in approximately 6 days. The rate of acquisition (mean increase in lever hits between sessions) was 1.4 ± 0.3. As no other studies reporting control values for Skinner box in rabbits could be found for comparison, we compared to control values reported in rats. In Wistar rats 79% of the tested animals achieved the learning criterion at a maximum of 7 days (Gallo et al., 1995), being the criterion 10 lever pressings with correct response in one session of 15 min. Another study in Wistar rats with a more demanding learning criterion (20 lever pressings with correct response in 15 min), described that controls needed approximately four sessions to reach it (Reyes-Castro et al., 2012). The heterogeneity of Skinner-box protocols must be taken into account when comparing results, and for example, the learning criterion has to be adapted to the species of work. In our protocol, if a learning criterion of 10 lever presses with correct response in one session would have been established, only 35% of the animals would have achieved it (again taking the litter as a statistical unit). Differences, not only in the learning criterion but also in the reward offered make it difficult, in general, to compare between studies.

On the contrary, the open-field test has been applied to rabbits in several other studies from different groups. In our case, latency time (calculated as the time in seconds the animal needs to leave the familiar starting point and start exploring the open field) was above 100 s while in many other studies it was less than 50 s (Gümüş et al., 2018; Van der Veeken et al., 2019, 2020). This is probably related to the fact that our protocol does not include a habituation session before the performance of the test. Total distance was very high compared to Van der Veeken et al. (2020) (ca. 1,600 cm), Van der Veeken et al. (2019) (ca. 1,400 cm) or Gümüş et al. (2018) (ca. 800 cm), but the mean time spent in the central area was similar to the one reported in (Van der Veeken et al., 2019) and was in accordance to the observed range in (Gümüş et al., 2018). The object recognition task has also been performed in rabbits by different groups but some of them perform it using visual objects (Hoffman et al., 2010; Hoffman and Basurto, 2013). This testing modality is also considered valid for assessing object recognition (Gümüş et al., 2018). However, some studies report that rabbits may be able to distinguish a novel visual object after a 5 min inter-trial interval, but not after 20, 180, or 360 min (Hoffman et al., 2010; Hoffman and Basurto, 2013), whereas in our studies discrimination with olfactory stimuli is present after a 30 min inter-trial interval (Illa et al., 2013) and this result could be replicated by another group (Gümüş et al., 2018).

The evaluation of plasticity-related endpoints on PND70 was performed in the hippocampus: the density of dendritic spines was measured in the hippocampal CA1 area, while the CA3 area was preferred for the analysis of perineuronal nets since a greater amount of *Wisteria floribunda* staining was observed in comparison to the CA1 (Illa et al., 2018), in agreement with previous works (Brückner et al., 2003; Hylin et al., 2013). The mean number of dendritic spines/µm obtained in the controls (1.8; Table 9) was very similar to the one previously reported by another research group in newborn rabbits (1.4, calculated from 67.8 spines in 50 µm in Balakrishnan et al., 2013). The number of contacts/µm^2^ of the extracellular matrix surrounding neurons, also referred to as perineuronal nets could not be compared to previous works, since, to the best of our knowledge, this endpoint was not evaluated before in rabbits.

In general, the evaluation of the results taking the litter as a statistical unit brings very similar results to those obtained taking the pup as a statistical unit in the controls. The standard deviations calculated for results obtained on PND1 were in all cases equal or smaller in case the litter was used as a statistical unit, but this was not the case for results obtained from PND50 on. However, in developmental neurotoxicity studies, to avoid previously described effects of serious exaggeration of significant effects in treated groups (Haseman and Hogan, 1975), the statistical unit of measure should be the litter and not the pup as also recommended in OECD TG 426 (OECD, 2007). For further discussion about this, the authors are referred to Harry et al. (2022), and articles included in this special issue.

## Discussion

Here, we present for the first time a comprehensive compilation of detailed protocols for the evaluation of neurodevelopmental alterations in the rabbit species. The protocols included covering relevant endpoints for developmental neurotoxicity assessment included in OECD TG 426 (OECD, 2007), which consists of “observations to detect gross neurologic and behavioral abnormalities, including the assessment of physical development, behavioral ontogeny, motor activity, motor and sensory function, and learning and memory; and the evaluation of brain weights and neuropathology during postnatal development and adulthood”. In addition, *in vitro* procedures to test developmental neurotoxicity effects in the rabbit species and to investigate their mechanism/mode of action have also been proposed in a way that the sample obtained for these procedures does not alter the integrity of the *in vivo* procedures.

Comparing the protocols included here with the tests requested in OECD TG 426 (OECD, 2007), the behavioral ontogeny evaluation proposed (Protocol 4) is much more comprehensive than the required one in OECD TG 426 (OECD, 2007), which only includes righting reflex, negative geotaxis, and motor activity. We include, as requested in the TG, protocols to evaluate neurologic adverse effects at different developmental time points, in our case one at the early postnatal period (PND 1) and one for young adults (around PND 70). In addition, the protocols presented allow, as recommended, the evaluation of relationships, in case they are present, between neuropathological and behavioral alterations at these different time points. However, in our battery we do not include a protocol to evaluate neuropathology in the PNS at a young adult stage, general neurobehavior assessment (FOB/Irwin test), or sexual maturation, and these assessments are required in OECD TG 426 (OECD, 2007). For articles describing the right endpoints and timings for sexual maturation evaluation in rabbits, the reader is referred to the work of Laffan et al. (2018) for female evaluation and the work of García-Tomás et al. (2009) for male evaluation. Also, in case more complex learning and memory tasks need to be evaluated, the reader is referred to previous descriptions of fear conditioning testing in rabbits (Chisholm and Moore, 1970; Supple et al., 1993). Concerning the analysis and interpretation of the results of the protocols, we have presented results analyzed in two different ways, one considering the litter and another one considering the pup/hemisphere/neuron as a statistical unit. In the present study, these two ways of analyzing the data have had minimal differences in the mean values of control animals and the standard deviations we obtained from these calculations were, in general, smaller when the litter was considered as the statistical unit (except for some of the Skinner test and open field results). However, when testing for developmental neurotoxicity, as indicated in the OECD TG 246 (OECD, 2007), littermates should not be treated as independent observations. This is because litter effects have been shown to exist and they can have a high impact in a toxicological study if the pup is taken as a statistical unit. Therefore, to avoid false-positive results, the statistical unit of measure should be the litter and not the pup (Abbey and Howard, 1973; Haseman and Hogan, 1975; Nelson et al., 1985; Holson and Pearce, 1992).

All tests presented in this collection have been used to evaluate the neurodevelopmental alterations induced by mild-hypoxic conditions during the prenatal period and some of them have been used by other authors to study the neurodevelopmental effects of caffeine exposure or maternal endotoxin exposure (Balakrishnan et al., 2013; Van der Veeken et al., 2020). The whole battery of tests has not been used to assess known neurotoxic compounds so far, but a broad definition of teratogen includes “any infection, physical, chemical, or environmental agent that can disrupt or disturb the development of a fetus or embryo (Adam, 2012)”, and in fact, hypoxia is accepted as a teratogen agent (Adam et al., 2021). Hypoxia, or low oxygen levels, is a neurodevelopmental key event that can be triggered by multiple causes, including a reduction or lack of blood flow (as presented here), low oxygen levels in the blood, low levels of red blood cells and/or hemoglobin, but also by the inability of the tissues to utilize oxygen due to, for example, carbon monoxide poisoning (Adam et al., 2021). Therefore, as an illustrative case study, the results of this battery of tests after chronic hypoxia-ischemia insult during gestation induced in one of the uterus horns by ligation of 40–50% of uteroplacental vessels on day 25 of pregnancy are discussed here, to show the potential of the combination of the different tests described in these protocols.

According to Nalivaeva et al. (2018), prenatal hypoxia in critical periods of neurodevelopment induces significant changes in cognitive functions at different postnatal stages which correlate with morphological changes in brain structures involved in learning and memory. The use of the battery of protocols proposed here allowed to detect learning and memory alterations in rabbits, since Skinner test results showed a lower proportion of cases reaching the learning criteria when compared with their controls (30 vs. 77%, *p* = 0.03, cases vs. controls, respectively; (Illa et al., 2017)) and a decreased discrimination index was observed in cases compared to controls when ORT was assessed (Illa et al., 2013; Illa et al., 2017). Morphological changes correlating with these findings were that cases presented a significant decrease in dendritic spines density and perineuronal nets immunoreactivity in the hippocampus when compared to controls (Illa et al., 2018). Even though the histological analysis presented here can be adapted to the region of interest for each study, in the results section we present the outcomes from the experimental design used to evaluate the effects of a chronic hypoxia-ischemia insult (Illa et al., 2018; Pla et al., 2021), which was selected in accordance to the degree of maturation and the susceptibility to hypoxic-ischemic events. The selected regions in the brain in the rabbit model are consistent with the ones chosen in other models such as rats (Back et al., 2002; Ruff et al., 2017), as detailed in each results section. However, the OECD TG 426 (OECD, 2007) indicates that tissue samples for the neuropathological examination should be representative of all major brain regions at PND 22 or earlier (as also recommended by Rao et al. (2011)), and also include samples from the spinal cord and PNS at study termination.

Additional information obtained at the functional level was that cases showed poorer results than controls in several of the endpoints assessed in the behavior ontogeny test at PND 1, including righting reflex, circular motion, intensity, sucking and swallowing, and head-turning (Pla et al., 2021). These findings were in accordance with decreased oligodendrogenesis observed in brain samples (Pla et al., 2021) as well as in neurospheres generated from these animals (Barenys et al., 2021), which could be related to this delay in the behavior ontogeny. The good correlation of the *in vivo* and *in vitro* findings indicates the added value of including protocols 2 and 3 in the battery.

Other functional deficits were also present at later time-points when cases presented a significantly increased latency of leaving the familiar starting point and a reduced number of external and internal boxes explored in the open field test at PND 70 (Illa et al., 2013; Illa et al., 2017). This result indicated that no motor problem was present in these animals, but they presented a higher degree of anxiety.

Considering all these results, we can distinguish a pattern of mild short-term impairments in motor, reflex and sensitivity and a long-term group of moderate alterations in learning, memory, and anxiety.

With all the information obtained, the theoretical benefits already mentioned in the introduction of having a rabbit developmental neurotoxicity model can also be discussed. The protocols included here take into account the higher similarity of rabbits to humans than rodent species regarding white matter maturation-timing and include endpoints that can measure alterations on it (such as astrocytes or oligodendrocyte IHC in Protocols 7 and 9 or reflex ontogeny in Protocol 4). Moreover, we have shown that the model can reflect neurodevelopmental alterations related to circulatory alterations, another aspect in which rabbits are more similar to humans than rodents (Foote and Carney, 2000; Carter, 2007; Eixarch et al., 2009). Considering these points, it is reasonable to think that for certain compounds or compound classes expected to cause neurodevelopmental alterations related to white matter alterations, or related to circulatory changes, the rabbit model could be useful for screening purposes, since it would be expected to better predict the human response. Other possible application scenarios would be in cases where the metabolism in rabbits is more similar to humans than in rodents, or in situations where a previous rabbit study raised concerns about neurotoxic effects (for example an OECD TG 414 study where the rabbit is the preferred non-rodent species). In this case, the recommendation of the OECD TG 426 is that the study is conducted in the species increasing the concern, but so far this could not be completely carried out if the concern was raised in rabbits because there were no protocols available. Some specific examples of compounds displaying structural-developmental neurotoxicity in rabbits and not in rats have already been identified (Theunissen et al., 2016; Teixidó et al., 2018). Unfortunately, one of the studies is based on a coded dataset that did not reveal the identity of the compounds showing species differences, but the identified substances (and the adverse neurodevelopmental effect) were: 10224 (small or absent cerebrum), 10330 (enlarged cerebral ventricle), and tafluprost (cranial and spinal malformations). Among the limitations of the protocols described here, we have to mention that the relevance of *in vitro* results in Protocol 3 to *in vivo* complex alterations such as learning or memory changes needs further assessment. Another limitation is that with the microglial protocol (Protocol 8) one can evaluate the density of microglia in a selected area, but it is not possible to evaluate the morphology of the cells. Similarly, for the evaluation of the perineuronal nets (Protocol 14), other studies include more comprehensive evaluations of structural alterations of perineuronal nets such as the number of perineuronal nets units, area, mean intensity of perineuronal nets marker expression, and shape parameters of perineuronal nets (Kaushik et al., 2021). Another limitation is the very high variability in the results of the total distance in the open field test, but other studies have reported high variabilities in this endpoint as well (Van der Veeken et al., 2020), which might indicate that other assessments within this assay, such as latency time or percentage of time spent in the central area, might be more useful to distinguish alterations in this test in rabbits.

However, should the proposed protocols be used under OECD TG 426 in the future, other important needs have to be addressed before regulatory acceptance: some required endpoints are not included in the approach: the minimum number of animals to be assigned to each group has to be clarified, the methods need to be validated and a higher number of studies should be evaluated to obtain more robust historical control values in this species.

As a final remark, the predictivity and sensitivity of this battery of tests for the assessment of developmental neurotoxicity in the rabbit species still need to be clarified, but this first approach of protocols adaptation is already a valuable tool for all research groups in need to study neurodevelopment in the rabbit species in toxicology and the first step to a possible future application under OECD TG 426.

## Data Availability

The original contributions presented in the study are included in the article/Supplementary Material; further inquiries can be directed to the corresponding author.

## Glossary

AU: airy unit
BMP7: bone morphogenetic protein 7
BSA: bovine serum albumin
CTB: CellTiter Blue
DAPI: 4′,6-diamidino-2-phenylindole
dH_2_O: distilled water
DIC: differential interference contrast
DMEM: Dulbecco’s modified Eagle’s medium
DMSO: dimethyl sulfoxide
DNT: developmental neurotoxicity
ECM: extracellular matrix
EGF: epidermal growth factor
FBS: fetal bovine serum
rhFGF: human recombinant fibroblast growth factor
FR: fixed ratio
GD: gestational day
GFAP: glial fibrillary acidic protein
HIER: heat-induced epitope retrieval
IHC: immunohistochemistry
MEM: minimum essential medium
NPC: neural progenitor cells
OECD: Organisation for Economic Co-operation and Development
o.n.: overnight
PBS: phosphate-buffered saline
PBST: PBS-triton X-100
PDL: poly-D-lysin
PFA: paraformaldehyde
PND: postnatal day
PNS: peripheral nervous system
Poly-HEMA: Poly(2-hydroxyethyl methacrylate)
rcf: relative centrifugal force
RT: room temperature
