# Supplementary material for: Protocols for the Evaluation of Neurodevelopmental Alterations in Rabbit Models In Vitro and In Vivo
Source: Front Toxicol. 2022 Jul 22;4:918520. doi: 10.3389/ftox.2022.918520 (PMC9355155; doi:10.3389/ftox.2022.918520)
Supplement: Supplementary file 1 [file DataSheet2.pdf]

BEHAVIORAL ONTOGENY TEST

**Fetus ID:** |_____________| - |__|__|__|__|__|	**Chip code/ear code** |__|__|__|__|__|	**Postnatal day:** |__|__|
**Date of evaluation:** |__|__| |__|__| |__|__|	**Exposure:** __________________	**Dose:** __________________

NEUROLOGICAL EVALUATION *(Derrick 2004) (0: worst – 3/4: best)* **Weigh fetus at +1P:** \|__\|__\|,\|__\|__\|g

**A- Posture**		**F- Intensity**	
0	Lays supine	0	No movement
1	Lays on side	1	Slight activity
2	Cannot maintain prone position	2	Distinct forceful movements
3	Prone position	3	Rapid forceful movements
**B- Righting reflex (10 tries)**		**G- Duration (observation for 1 minut)**	
	Nº times turns prone from supine |__|rapid	0	No movement
**C- Tone**		1	Activity <20 sec
0	No increase in tone	2	Activity 20-40 sec
1	Slight increase in tone when limb is moved	3	Activity >40 sec
2	Marked increase in tone but limb is easily flexed	**H- Lineal movement (1min, distance 15 cm)**	
3	Increase in tone, passive movement difficult		Nº of times crosses perpendicular line
4	Limb rigid in flexion or extension	**I- Shortest fore-hindpaw distance**	
**D- Circular motion (one minut)**			5 measurements (if pup walks in straight line)
0	No movement	**J- Sucking and swallowing**	
1	Slight movement, slight jump	0	No movement of jaw, milks dribbles out completly
2	Good range of motion, maintains for 1or 2 steps occasional jump	1	Some movement of jaw and neck, most of milk dribbles out
3	Entire range of motion, at least 3 steps,rapid jumps	2	Definite suck and swallow, some milk in nose
**E- Locomotion:**	Head | Forelimbs | Hindlimbs	3	Good suck and swallow, no milk in nose
0	No movement |__| |__| |__|	**K- Head turning during feeding**	
1	Slight movement |__| |__| |__|	0	No movement
2	Distinct movement, |__| |__| |__|	1	Slight occasional movement of head
3	Rapid movement, |__| |__| |__|	2	Distinct movement of head
		3	Rapid forceful movements of head and body

**ODOUR TEST**	Aversive response |_____|	(0-No,1-subtil, 2-low response, 3-correct)	Latency in aversive response |___| (seconds)
