# Supplementary material for: Protocols for the Evaluation of Neurodevelopmental Alterations in Rabbit Models In Vitro and In Vivo
Source: Front Toxicol. 2022 Jul 22;4:918520. doi: 10.3389/ftox.2022.918520 (PMC9355155; doi:10.3389/ftox.2022.918520)
Supplement: Supplementary file 2 [file DataSheet1.pdf]

Supplementary file 1. REAGENTS AND SOLUTIONS

Reagents and solutions protocol 1

a) Medication for anesthetic induction

i. Ketamine 50 mg/dl (35 mg/kg) – total volume calculation: mg/50mg

ii. Xylazine 2% (5 mg/kg) – total volume calculation: mg/20 mg

iii. Atropine: 1.0 - 3.0 mg/kg

b) Medication for anesthetic maintenance

i. Put 4 ml of ketamine and 3 ml of xylacine in 100 ml of FS. Connect FS with serum equipment and connect to a high-precision flow regulator, adjust it to 30 – 40 ml/h (necessary dose for an animal of 4-5 kg)

ii. For rescue anesthesia: syringe with propofol 10 mg/kg e.v. (never > 15 – 20 mg/kg)

Reagents and solutions for protocol 2

a) Tissue digestion solution:

i. 975 µL MEM (prewarmed).

ii. 20 µL Papain.

iii. Incubate 10 min at 37 ºC.

iv. Add 10 µL DNase I.

v. Sterile filtrate with a syringe filter.

b) Ovomucoid solution:

i. 975 µL DMEM (prewarmed).

ii. 10 µL DNase I.

iii. 5 µL BSA (10 % in PBS).

iv. 10 µL Trypsin-Inhibitor (prewarmed).

v. Sterile filtrate with syringe filter.

c) Freezing medium:

i. 70 % B27.

ii. 20 % FBS.

iii. 10 % DMSO.

d) Poly-HEMA coating:

i. Materials:

- Poly (2-hydroxyethyl methacrylate).

- 96 % (v/v) ethanol.

- H_2O, deionized and sterile.

ii. Poly-HEMA solution:

- Pour 39.5 mL of 96 % (v/v) ethanol and 500 µL of $_{d}H_2O$ into a 50 mL conical tube and mix them.

- After mixing, add 1.2 g of poly-HEMA into the conical tube and dissolve it using a plate rotator o.n. at RT.

- Store this poly-HEMA stock solution at 4 ºC for up to 2 months.

iii. Coating of dishes and plates with Poly-HEMA solution:

- Apply the appropriate volume of the poly-HEMA solution (Table S1) to each dish or well in the tissue culture hood.

- Spread the poly-HEMA solution over the entire surface of each dish or well.

- Leave the dish or plate o.n. without lid to allow the poly-HEMA solution to completely evaporate.

- The poly-HEMA–coated dishes can be used for 3 months after coating when stored at RT in the dark.

Table S1. Volume of poly-HEMA solution for dish or well coating.

Dish size	Volume of poly-HEMA solution
90 mm (diameter)	3.2 mL
60 mm (diameter)	1.3 mL
35 mm (diameter)	500 µL
12 well	200 µL
24 well	100 µL
48 well	70 µL
96 well	25 µL

Reagents and solutions for protocol 3

a) Laminin solution: Add $_{d}H_2O$ to the Laminin solution to reach a final concentration of 1 mg/mL. The amount of water added needs to be determined for each new lot. Store Laminin at -20 ºC.

b) PDL solution: Dilute 50 mg PDL in 500 mL $_{d}H_2O$, aliquot and store at -20 ºC.

c) First antibody solution immunocytostaining of neurospheres:

i. 1st antibody (dilution as indicated in Table S2).

ii. 10 % goat serum.

iii. PBS or PBST for intracellular epitopes.

d) Second antibody solution immunocytostaining of neurospheres:

i. 2nd antibody (dilution as indicated in Table S2).

ii. 1 % Hoechst (stock: 0.2 mg/mL).

iii. 2 % goat serum.

iv. PBS.

Table S2. Antibody solutions

1st antibody		Species	Type	Antigene	Dilution	Incubation
O4	Oligodendrocytes	Mouse	IgM	Surface epitope	1:200	o.n., 4 ºC
β(III)-Tubulin	Neurons	Rabbit	IgG	Cytoskeleton	1:200	1h, 37 ºC
GFAP	Astrocytes	Rabbit	IgG	Cytoskeleton	1:200	o.n., 4 ºC
2nd antibody		Species	Type	1st antibody binding	Dilution	Incubation
anti-Mouse IgM, Alexa Fluor 488		Goat	IgM	O4	1:200	30 min, 37 ºC
anti-Rabbit IgG, Alexa Fluor 488		Goat	IgG	β(III)-Tubulin	1:100	30 min, 37 ºC
anti-Rabbit IgG, Alexa Fluor 546		Goat	IgG	GFAP	1:200	30 min, 37 ºC

Abbreviations: o.n.: overnight.

e) PDL-laminin coating of 8-Chambered Cell Culture Slides:

i. Thaw PDL solution at 37 ºC and add 250 µL PDL solution to chambers of an 8-chamber slide.

ii. Incubate for 1 h at 37 ºC.

iii. Thaw Laminin solution at 4 ºC and dilute it 1:100 in $_dH_2O$.

iv. Remove PDL solution.

v. Wash chambers/wells with $_dH_2O$.

vi. Add laminin dilution to chambers/wells.

vii. Incubate for 1 h at 37 ºC.

viii. Wash chambers with $_dH_2O$ and sterile PBS.

ix. Coated slides can be stored at 4 ºC for up to one week.

Table S3. Volume of PDL and Laminin solution for dish or well coating.

**Dish size**	**PDL and Laminin**	**H_2O washing and PBS storage**
8-chamber-slide	250 µL	500 µL
96 well	50 µL	100 µL
48 well	150 µL	300 µL
24 well	300 µL	500 µL
12 well	500 µL	1 mL
6 well	1 mL	2 mL

f) Controls for the neurosphere differentiation and migration assays:

i. Background control: only N2 media without spheres.

ii. Solvent control: N2 media with respective solvent with spheres.

iii. Positive controls:

- Lysis control: N2 media with respective solvent with spheres.

- Oligodendrocyte differentiation: 100 ng/mL BMP7.

- Neuronal differentiation: 10 ng/mL EGF.

- Migration: 10 µM PP2.

g) Controls for cell titer blue (CTB) assay:

i. Lysis control: culture medium with cells. Add DMSO to a final concentration of 10% 30 min before addition of CTB-reagent.

ii. Background control: culture medium without cells.

h) Controls for proliferation assay:

i. Solvent control: B27 media with respective solvent.

ii. Positive control: B27 media without growth factors (B27 w/o).

iii. If combined with CTB Assay prepare lysis control and background control.

- Background control: only B27 media.

- Lysis control: Solvent control with spheres.

Reagents and solutions for protocol 5

a) Sucrose 30 %: 30 g sucrose in 100 mL phosphate buffer 0.1 M.

Reagents and solutions for protocol 6

a) Solution A+B in proportion 1:1 (should be prepared 24 h prior to use, and has 1 month of viability). Prepare 5 mL of solution/1 cm^3 of tissue (15 mL per brain, approximately).

b) Cresyl violet: 0.1 g/100 mL $_dH_2O$ + 2 drops of acetic acid. Prepare 24 h before use, viability 1 year.

Reagents and solutions for protocols 7, 8, 9 and 14.

a) IHC blocking solution:

i. 10 % fetal bovine serum (10 mL/100 mL).

ii. 0.2 M glycin (0.15 g/100 mL).

iii. 0.2 M gelatin (0.2 g/100 mL).

iv. PBST 0.3 %.

b) PBST 0.3 %: 2.4 mL Triton + 797.6 mL PBS.

c) Oligodendrocyte immunocytochemistry solutions:

i. Primary antibody (Mouse IgM anti-O4) solution: 1 µL antibody + 49 µL PBST/cut.

ii. Secondary antibody (Alexa Fluor 488 goat anti-mouse IgM) solution: 0.5 µL Hoechst + 49,375 µL PBST + 0.125 µL antibody/cut.

d) Perineuronal nets immunocytochemistry solutions:

i. Primary antibody solution: 20 µL antibody + 190 µL blocking solution + 190 µL PBST 0.3 % /slide.
